# Efficient photocatalytic hydrogen evolution with ligand engineered all-inorganic InP and InP/ZnS colloidal quantum dots

**DOI:** 10.1038/s41467-018-06294-y

**Published:** 2018-10-01

**Authors:** Shan Yu, Xiang-Bing Fan, Xian Wang, Jingguo Li, Qian Zhang, Andong Xia, Shiqian Wei, Li-Zhu Wu, Ying Zhou, Greta R. Patzke

**Affiliations:** 1 School of Materials Science and Engineering, Southwest Petroleum University, No. 8, Xindu Road, Xindu District, Chengdu, 610500 China; 2 Department of Chemistry, University of Zurich, Winterthurerstrasse 190, CH-8057 Zurich, Switzerland; 3 Key Laboratory of Photochemical Conversion and Optoelectronic Materials, Technical Institute of Physics and Chemistry, Chinese Academy of Sciences, Beijing, 100190 China; 4 Beijing National Laboratory for Molecular Sciences, Key Laboratory of Photochemistry, Institute of Chemistry, Chinese Academy of Sciences, Beijing, 100190 China

## Abstract

Photocatalytic hydrogen evolution is a promising technique for the direct conversion of solar energy into chemical fuels. Colloidal quantum dots with tunable band gap and versatile surface properties remain among the most prominent targets in photocatalysis despite their frequent toxicity, which is detrimental for environmentally friendly technological implementations. In the present work, all-inorganic sulfide-capped InP and InP/ZnS quantum dots are introduced as competitive and far less toxic alternatives for photocatalytic hydrogen evolution in aqueous solution, reaching turnover numbers up to 128,000 based on quantum dots with a maximum internal quantum yield of 31%. In addition to the favorable band gap of InP quantum dots, in-depth studies show that the high efficiency also arises from successful ligand engineering with sulfide ions. Due to their small size and outstanding hole capture properties, sulfide ions effectively extract holes from quantum dots for exciton separation and decrease the physical and electrical barriers for charge transfer.

## Abstract (web summary)

While quantum dots show high efficiency solar-to-fuel conversion for renewable energy, the frequently toxic elements employed present severe safety concerns. Here, authors demonstrate indium phosphide quantum dots as low-toxicity alternatives alongside efficient hydrogen evolution photocatalysis.

## Introduction

Conversion of solar energy into storable hydrogen fuel through water splitting is one of the most sustainable solutions to meet the increasing worldwide energy demands^1,2^. Among the wide spectrum of photosensitizer types that has emerged over the past years^3–8^, colloidal quantum dots (QDs) stand out through combining molecular versatility with the efficient light harvesting properties of semiconductors^9,10^, and facile charge carrier transport through interfaces^10,11^. Photocatalytic processes with semiconductor nanomaterials and QDs^12,13^, such as CdS, do not only provide most direct low-tech access to clean hydrogen, but also permit fundamentally new insight into the photophysics of hydrogen production^14^. CdS/CdO_x_ QDs furthermore open up new avenues for solar photoreforming of lignocellulose biomass to hydrogen^15^. To date, Cd-chalcogen II–IV QDs remain the most efficient QD types in photocatalytic systems^15–20^. However, the toxicity of Cd severely limits their large-scale application options, not only in photocatalytic water splitting, but also in other urgent global challenges, such as water disinfection^21^ and the elimination of multidrug-resistant bacteria^22^ with photoexcited QDs. This current need for efficient non-toxic QDs has brought forward complex engineering approaches towards multinary I–III–VI_2_ and I_2_–II–IV–VI_4_ nanoscale semiconductors^23^.

InP QDs are an attractive alternative target for direct solar-to-fuel conversion. Furthermore, InP is well suited for environmental and biological applications^24^, and the notably lower toxicity of InP/ZnS QDs compared to CdSe/ZnS QDs has recently been demonstrated both in vitro and in vivo^25^. Surprisingly, however, InP QDs have rarely been studied for their artificial photosynthesis properties, although InP nanomaterials were used in breakthrough photoelectrode and solar cell studies^26,27^. In comparison to the most widely investigated CdSe QDs, InP has a smaller bulk band gap (1.35 eV vs 1.76 eV) and larger Bohr exciton radius (9.6 nm vs 4.6 nm), which widens its corresponding absorption and emission range. In addition, InP displays a more evident extent of covalent bonding than CdSe, leading to higher delocalization of wavefunctions and weaker attraction between electrons and holes, as well as weaker phonon coupling^24^.

Here we show the potential of InP QDs as hydrogen evolution photosensitizers. Inspired by recent synthetic developments^28^, we access a series of InP and InP/ZnS QDs using economic and safe (dialkylamino)phosphines as phosphorus source. Modification of the as prepared QDs with inorganic sulfide (S^2-^) ions^29,30^ furthermore brings forward progress in ligand engineering of QDs, as a key area in solar energy conversion^31^. These sulfide ions not only endow QDs with good water solubility, but also with remarkable activity in photocatalytic processes^32^. We demonstrate the important role of S^2−^ ligands for photocatalytic hydrogen evolution by comparing different organic and inorganic ligand types. S^2−^ ligands not only effectively facilitate the extraction of photogenerated holes from the QDs to the surface ligands, but also decrease the charge transfer barriers between QDs and their surrounding acceptors, thus notably enhancing photocatalytic hydrogen evolution.

## Results

### Synthesis and characterization

**Fig. 1:**
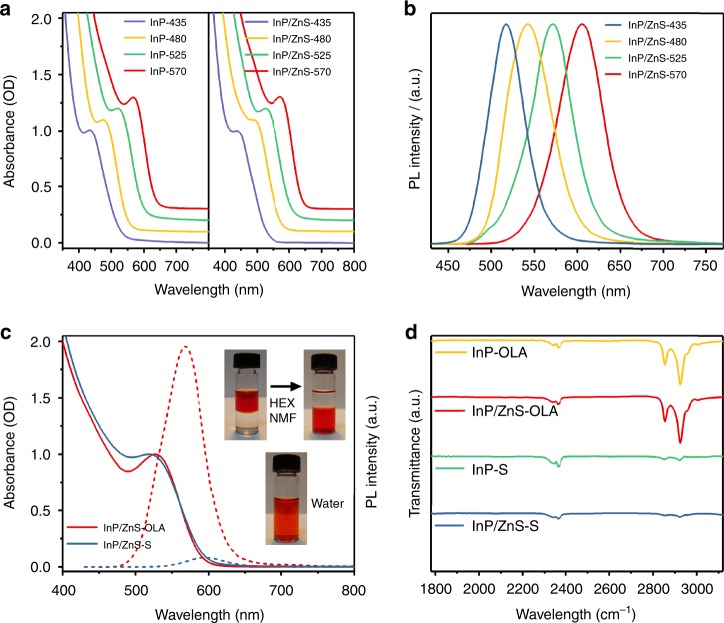
Spectroscopic characterization of InP and InP/ZnS QDs. **a** Absorption spectra of different InP and InP/ZnS (15 min) QDs in hexane. **b** PL spectra of different InP/ZnS (15 min) QDs in hexane. **c** Absorption spectra and PL spectra before and after ligand exchange for InP/ZnS QDs (525 nm, 15 min) in hexane and water (the absorbance of the two QD types was adjusted to the same value at the excitation wavelength to enable PL comparison). **d** FT-IR spectra of QDs before and after ligand exchange

InP and InP/ZnS colloidal QDs were synthesized based on a modified literature protocol (see Supplementary Fig. 1 for photographs)^28,33^. First, the size of the QDs was adjusted by tuning the ratio of ZnCl_2_ to ZnI_2_, instead of introducing of different indium halides. Furthermore, the growth temperature for ZnS was directly held constant at a higher value prior to the addition of sulfur (cf. Methods, Supplementary Method 1 and Supplementary Note 1 for further information). The characteristic absorption peak of the QDs undergoes a blue shift from 570 nm to 435 nm upon increasing the ratio of ZnI_2_ to ZnCl_2_ in the system (Fig. 1a). Likewise, the emission peak of InP/ZnS QDs is shifted from 605 nm to 515 nm (Fig. 1b). The typical emission spectra of the as-synthesized InP/ZnS QDs exhibit FWHM (full width at half maximum) values around 60 nm, close to the reported optimal results^33–35^. While coating of InP QDs with ZnS barely changes the absorption spectrum of the original InP QDs, it leads to a significant improvement of the photoluminescence quantum yield (PLQY) of the system from 1.0 to 46% with increasing growth time of the capping layer (Supplementary Fig. 2). This confirms that the growth of ZnS on QDs efficiently passivates their surfaces^36^.

Ligand exchange was then carried out to replace the original long-chain organic ligands (oleylamine, OLA) on the surface of the QDs (referred to QDs-OLA) with inorganic S^2-^ (referred to QDs-S; cf. Methods for details)^30^. The success of this process is evident from the efficient phase transfer of the treated QDs from hexane to *N*-Methylformamide (NMF) (inset of Fig. 1c). These S^2-^ capped QDs could be well dissolved in water by further precipitation and redispersion, in line with their sufficiently negative zeta potential (Supplementary Fig. 3). The excitonic features in the absorption spectra remain constant after ligand exchange, implying that the size changes of the QDs are negligible after sulfide capping (Fig. 1c and Supplementary Fig. 4)^30,37^. Nevertheless, the PLQY of InP/ZnS QDs after ligand exchange dropped drastically (Fig. 1c) due to the increased amount of surface traps and nonradiative relaxation processes^38,39^. Efficient ligand exchange is furthermore evident from the almost complete disappearance of the ligand C-H stretching FT-IR bands at 2855 and 2924 cm^−1^ (Fig. 1d)^29,30^. In addition, thermogravimetric analysis (TGA) of S^2−^ capped QDs shows no obvious weight loss between 100 and 500 °C in contrast to ligand loss of OLA capped QDs (Supplementary Fig. 5), thus providing further evidence for the removal of most organic ligands^29,37^.

TEM and HRTEM images indicate a typical narrow size distribution of the InP QDs (525 nm) with an average size around 2.7 nm, which is close to literature data^28^. After growth of ZnS for 15 min, the size of QDs changed slightly (Supplementary Figs. 6 and 8). After ligand exchange, the shape and size of InP and InP/ZnS QDs did not change obviously (Supplementary Figs. 7 and 8), although a slight degree of aggregation took place due to the less repulsive effect of inorganic sulfide ions compared to long-chain organic molecules. Due to the very thin ZnS layer and the small size of QDs, the direct assignment of ZnS based on lattice planes or EDS elemental mapping is difficult. However, a less crystalline zone outside the InP core in the aberration-corrected STEM image (Supplementary Fig. 9) may arise from the surface modification of QDs with such a thin ZnS layer^40^. Powder X-ray diffraction (PXRD) data (Supplementary Fig. 10) confirm that the zinc blende structure of the QDs is maintained during both ZnS growth and ligand exchange processes, accompanied by a peak shift to higher angles from InP QDs to InP/ZnS QDs^28,35^. Elemental analyses by inductively coupled plasma optical emission spectrometry (ICP-OES, Supplementary Table 1) and energy-dispersive X-ray spectroscopy (SEM-EDS, Supplementary Table 2) both show that ZnS growth on InP QDs within 15 min is slow under the applied synthetic conditions, according to the low Zn/In atomic ratio in the QDs. Generally speaking, the notable lattice mismatch (7.7%) of ZnS and InP renders the growth of thick ZnS layers difficult^36^. Although ZnS layers obtained from longer reaction times would improve the PLQY of QDs (Supplementary Fig. 2b)^28^, it may not be an optimal choice for photocatalysis (see below). In addition, the sulfur content in the composites after ligand exchange increased obviously, which is in line with the above analysis.

### Photocatalytic hydrogen evolution

**Fig. 2:**
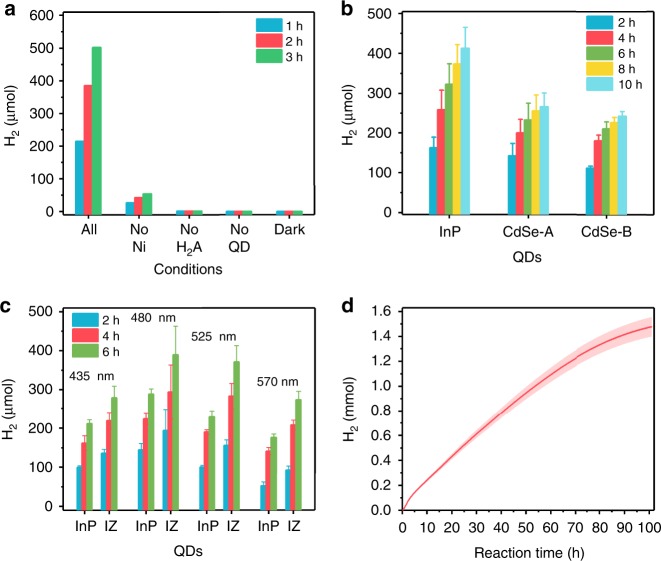
Photocatalytic hydrogen evolution experiments. **a** Amount of hydrogen evolved from reference systems without Ni^2+^, H_2_A, QDs or light illumination, respectively. **b** Comparison of hydrogen evolution results with InP-S QDs (525 nm) and two types of CdSe-S QDs (~525 nm). **c** Hydrogen evolution with InP-S QDs and InP/ZnS-S QDs (abbreviated as IZ) with different excitonic absorption peaks. **d** Long time hydrogen evolution of InP/ZnS-S QDs (shadowed area: error range). Conditions: **a** 1.6 μM InP/ZnS QDs, **b** 1.6 μM InP-S QDs (525 nm) and CdSe-S QDs with the same absorbance at 525 nm or **c** 2 μmol (of molecular concentration) QDs with different absorption peak, with 0.035 mM Ni^2+^ in 6 mL of 0.2 M H_2_A (pH 4.5) were illuminated with **a**, **b** LED light source (525 nm, 4 × 1 W) and **c** AM 1.5 simulator light; **d** 1.9 μM QDs (525 nm, 15 min) and 0.042 mM Ni^2+^ in 10 mL of 0.2 M H_2_A (pH 4.5) with LED light source (453 nm, 0.23 W). Error bars were estimated based on the standard deviation according to two or more independent experiments

After the concentration of the colloidal solution was determined (cf. Supplementary Method 2), InP-S and InP/ZnS-S QDs were tested for hydrogen evolution in the presence of ascorbic acid (H_2_A) as electron donor and Ni^2+^ as proton reduction catalyst, along the lines of the reported assays for Cd-based QDs^17,41^. Control experiments show that light, photoabsorber QDs, the electron donor H_2_A, and the Ni^2+^ catalyst are all indispensable for efficient hydrogen evolution (Fig. 2a), and the optimal concentration of Ni^2+^ and H_2_A as well as the optimal pH for the present system were studied (Supplementary Fig. 11). Moreover, we compared the photocatalytic performance with pristine InP-S QDs (525 nm) with two conventional CdSe-S QD batches from different protocols (with similar absorption peak, size, and ligand exchange procedures) under analogous conditions (see Supplementary Method 3). The results show that the hydrogen generation efficiency of InP QDs is even higher than that of CdSe QDs (Fig. 2b). This demonstrates that InP-based QDs bear a high potential for photocatalytic hydrogen evolution with environmentally friendly materials. Furthermore, the overall photocatalytic hydrogen evolution efficiency was enhanced after the growth of ZnS layers on InP QDs (Supplementary Fig. 12). In particular, InP/ZnS QDs with a ZnS growth time of 15 min (with a thin ZnS layer, see results above) outperform the other samples. As ZnS has a higher conduction band and a lower valence band, a type I heterostructure is formed between InP and ZnS. Therefore, electrons and holes tend to localize in InP. Although ZnS can decrease the concentration of some intrinsic surface defects, extraction of the photogenerated charges by Ni^2+^ and H_2_A would be less efficient in case of a thick ZnS layer^42^, which is in sharp contrast to the requirements for high PLQY. Moreover, hydrogen evolution was evaluated for InP and InP/ZnS QDs with different excitonic absorption peaks under illumination with an AM 1.5 solar simulator (Fig. 2c), and all of them exhibited good performance. This may result from their optimal band gap settings, which ensure both efficient absorption of the incident light from the solar simulator and the sufficient driving force for photogenerated charge transfer and subsequent reactions.

Under optimal conditions, photocatalytic hydrogen evolution was performed with different light sources (Supplementary Fig. 13). The hydrogen generation rate for the system reaches 45 mmol h^−1^ g^−1^ within 64.5 h (see Supplementary Fig. 13a and Supplementary Method 4) with a LED light source, and the TON value is as high as 128,000 based on InP/ZnS-S QDs (and 5900 normalized to Ni centers). The apparent quantum yield (AQY) of the system correlates well with the absorption spectra of InP/ZnS QDs, and the internal quantum yield (IQY) reaches 31% at 525 nm (Supplementary Method 5, Supplementary Table 3 and Supplementary Fig. 14). When using an AM 1.5 solar simulator as an alternative light source, the TON and hydrogen evolution rate within 20 h are 61,500 and 70 mmol h^−1^ g^−1^, respectively (Supplementary Fig. 13b). Online photocatalytic hydrogen evolution tests show that InP/ZnS-S QD systems display long-term activity over 100 h (Fig. 2d). PXRD patterns further confirm that the diffraction peaks of QDs are still distinguishable after evolution of 0.65 mmol H_2_ (Supplementary Fig. 15). The decline of the hydrogen evolution rate after long time irradiation may be caused by the accumulation of the oxidation product dehydroascorbic acid (DHA)^43^, as well as by partial degradation of the QDs. However, the total amount of H_2_ production is satisfactory and competitive in all of the investigated reaction conditions. The above results in their entirety clearly highlight S^2−^ capped InP and InP/ZnS QDs as a promising photosensitizer type.

### Photophysical study of the system

To study the mechanisms behind the photocatalytic process, the thermodynamic properties of InP/ZnS QDs were first evaluated. The conduction and valence band edge positions of InP/ZnS QDs were determined as −0.92 V and 1.35 V (vs NHE) through cyclic voltammetry (CV) measurements (Supplementary Fig. 16)^44^, in line with other reports^26^. Given the standard redox potentials of Ni^2+^/Ni (−0.25 V vs NHE) and H_2_A/DHA (0.36 V vs NHE)^41,45^, electron transfer from QDs to Ni^2+^ and hole transfer from QDs to H_2_A thus appears feasible. DHA hydrate is the main oxidation product of H_2_A after photocatalysis, which was verified through ^13^C NMR spectroscopy (Supplementary Fig. 17). However, the formation of metallic Ni was not detected by EPR spectroscopy of the present system after illumination (Supplementary Fig. 18), in line with some other reports^17,46^. In addition, no significant absorption changes were observed and the zeta potential of the system remained sufficiently negative at pH 4.5 (cf. Supplementary Fig. 19, Supplementary Note 2, and Supplementary Table 4). This indicates that the majority of the S^2−^ ligands on the QDs are retained under the photocatalytic conditions.

**Fig. 3:**
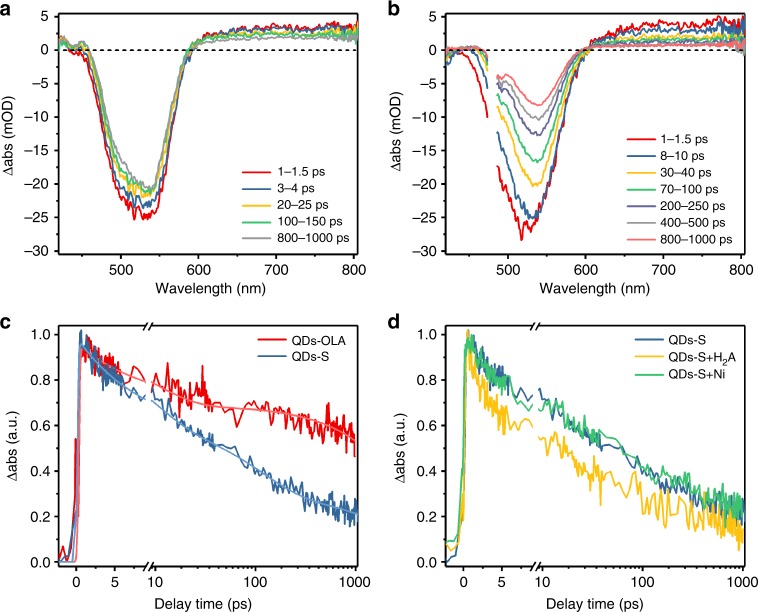
fs-TA spectra of InP/ZnS QDs (525 nm, 15 min). **a** TA spectra of InP/ZnS-OLA QDs; **b** TA spectra of InP/ZnS-S QDs at indicated delay time windows. **c** TA kinetics at 725 nm (average from 700 nm to 750 nm) for the PA signal and the corresponding fitting curve of InP/ZnS-OLA and InP/ZnS-S QDs. **d** TA kinetics at 725 nm (average from 700 nm to 750 nm) for the PA signal of InP/ZnS-S QDs with and without introduction of H_2_A or Ni^2+^ into the solution. Conditions: pump pulses wavelength: 480 nm

Time-resolved spectroscopy was then performed to obtain direct insight into the photophysical behavior of charge carriers. Femtosecond transient absorption (fs-TA) experiments were firstly conducted using a home-built femtosecond broadband pump-probe setup with time resolution around 100 fs as described previously in detail^47^. Concentrations of samples were adjusted to an absorbance of 0.3 OD at a pumping wavelength of 480 nm in 1 mm pathlength quartz cuvettes, and continuous stirring was performed during the test to alleviate photodegradation. The TA spectra (Fig. 3a, b) display two prominent features, namely a typical excitation bleaching (XB) and a featureless broad photoinduced absorption (PA). The XB signal is usually attributed to the state filling of the 1 S electron level in the conduction band, as state filling of the 1 S hole level is often negligible due to a higher density and degeneracy of the hole levels in QDs^48,49^. In contrast, both electrons and holes can be responsible for the PA signal, and it may vary with different QDs and surface states^49,50^. We here analyzed the origin of PA in the present system with respect to two aspects. One is the different decay behavior for the kinetics for XB and PA (Supplementary Fig. 20). This means that the species contributing to PA differs from that responsible for XB^50^. The other strategy is the analysis of the kinetic changes after H_2_A and Ni^2+^ were introduced (Fig. 3d)^49,51^. When the electron donor H_2_A is added, the decay of PA becomes faster, while no obvious changes occur after addition of Ni^2+^. Therefore, we attribute the PA signals mostly to the holes in InP/ZnS QDs.

Based on this analysis, we further compared the kinetics for the XB (Supplementary Fig. 21a) and PA (Fig. 3c) signals of both InP/ZnS-OLA QDs and InP/ZnS-S QDs, respectively. For InP/ZnS-OLA QDs, the decay of TA signal is slow, indicating that the trap state within the QDs is suppressed, in line with their bright luminescence. After ligand exchange of OLA by S^2−^, both XB and PA decay become faster. Usually, S^2−^ ligands can act as hole traps above the valence band^38,52^. Although the hole transfer to S^2−^ seemingly should not affect the kinetics of XB, the faster recovery of XB is not surprising (Supplementary Fig. 21a), given that there are numerous works reporting that the hole transfer process indeed leads to a faster decay of XB, which is still subject to discussions^53–55^. In contrast, the faster decay of the PA signal for holes in InP/ZnS-S QDs can be clearly explained with the hole transfer process (Fig. 3c). Other than OLA, the S^2−^ ligands on the surface accept photogenerated holes from QDs, thus providing an additional pathway for hole extinction, which accelerates the disappearance of the hole related transient PA signal. Fitting of PA signal kinetics clearly illustrates that an additional decay process with a timescale around 110 ps appears for InP/ZnS-S QDs in comparison to InP/ZnS-OLA (Supplementary Table 5), which is consistent with the hole transfer process from QDs to S^2−^ ligands.

Moreover, comparison of the kinetic decay of InP/ZnS-S QDs in the presence/absence of Ni^2+^ or H_2_A demonstrates that charge transfer proceeds on the ps scale in the system. On the one hand, hole transfer is evident from the faster decay of the PA signal after the introduction of H_2_A into the solution (Fig. 3d); and on the other hand, electron transfer from QDs to Ni^2+^ is observable as well from the faster recovery of the XB electron signal after the addition of Ni^2+^catalyst (Supplementary Fig. 21b)^49,56^.

**Fig. 4:**
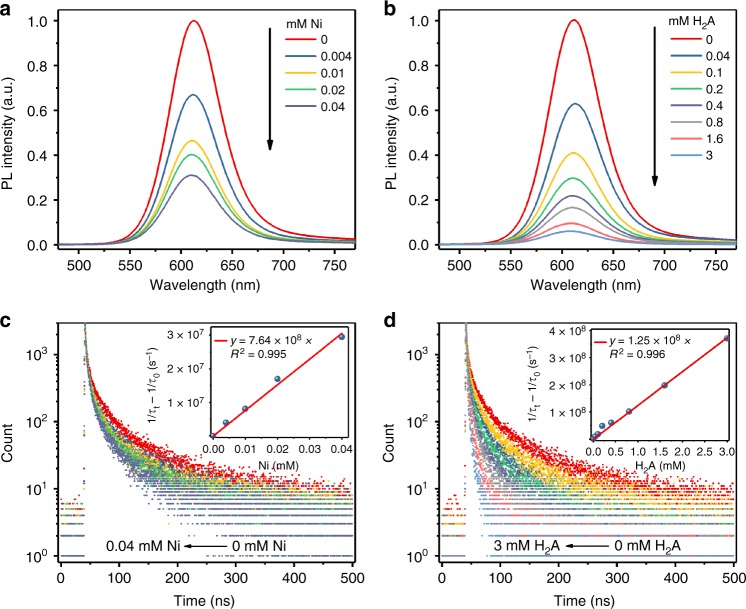
Steady state and time-resolved photoluminescence quenching experiment of InP/ZnS-S QDs (525 nm, 15 min). **a**, **b** Static photoluminescence quenching for InP/ZnS-S QDs in water after addition of different amounts of Ni^2+^ and H_2_A. **c**, **d** PL decay curves of InP/ZnS-S QDs after addition of different amounts of Ni^2+^ and H_2_A respectively; the inset shows the fitting curve for lifetime changes with increased concentration of Ni^2+^/H_2_A. Conditions: pH of all solutions was adjusted to 4.5; excitation wavelength: 406 nm

Due to the limited timescale of the fs-TA experiment (up to 1 ns), further information about the charge transfer processes on the nanosecond scale was obtained from steady-state and time-resolved emission spectra of InP/ZnS-S QDs. The emission of the QDs was quenched notably after introduction of small amounts of Ni^2+^ and H_2_A into the system, and this effect increased gradually with higher amounts (Fig. 4a, b). The kinetic emission decay of the QDs became faster as well, in line with the decrease of emission intensity (Fig. 4c, d). Fitting results show that the average lifetime of InP/ZnS QDs dropped from 37.0 ns to 17.7 ns and from 38.2 ns to 2.52 ns with Ni^2+^ and H_2_A concentrations of 0.04 mM and 3 mM, respectively (see Supplementary Tables 6 and 7 and Supplementary Method 6). This points to a calculated electron transfer rate from QDs to Ni^2+^ of 7.64 × 10^8^ s^−1^ mM^−1^ and a hole transfer rate from QDs to H_2_A of 1.25 × 10^8^ s^−1^ mM^−1^ (inset of Fig. 4c, d).

### Significance of sulfide capping

The above analysis indicates that the surface S^2−^ ligands influence the photophysical processes of QDs, which may also contribute to the high hydrogen evolution performance of the present system. We thus further explored the role of surface sulfide ligands beyond their endowing QDs with water solubility. To that end, we systematically compared InP/ZnS-S QDs to a series of QDs capped with other ligands with respect to their respective activity in photocatalytic hydrogen evolution. The ligands were selected with respect to two criteria. First the ligand exchange process should be easy and efficient, and secondly the ligands should stabilize InP and InP/ZnS QDs as colloids in solution.

**Fig. 5:**
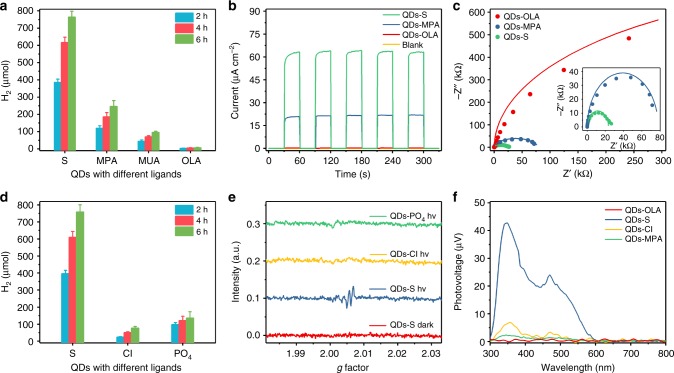
Influence of different ligands on the hydrogen evolution performance and photophysical properties of InP/ZnS QDs (525 nm, 15 min). **a** Hydrogen evolution for InP/ZnS QDs capped with different organic ligands. Conditions: 1.6 μM QDs and 0.035 mM Ni^2+^ in 6 mL of 0.2 M H_2_A (pH 4.5) were illuminated with a LED light source (525 nm, 4 × 1 W). **b** Transient photocurrent and **c** electrochemical impedance spectra of InP/ZnS QDs with different ligands at open circuit voltage under simulated sunlight illumination. Conditions: electrolyte: 0.2 M H_2_A, pH 4.5; frequency range: 0.1–10^5^ Hz. **d** Hydrogen evolution for InP/ZnS QDs capped with different inorganic ligands. Conditions: 1.6 μM QDs and 0.035 mM Ni^2+^ in 6 mL of 0.2 M H_2_A (pH 4.5) were illuminated with a LED light source (525 nm, 4 × 1 W). **e** EPR spectra for InP/ZnS QDs with different ligands. The magnetic field intensity was transformed into *g* factor values. **f** SPV spectra for InP/ZnS QDs capped with different ligands. Xe lamp was used for irradiation. Error bars were estimated based on the standard deviation according to three independent experiments

Usually, colloidal QDs synthesized in organic phases are capped with oil-soluble long alkyl chains (OLA in the present case), and the conventional ligands used to transfer QDs from nonpolar solvents to water are organic thiols. We thus prepared InP/ZnS QDs capped with 3-mercaptopropionic acid (MPA) and 11-mercaptoundecanoic acid (MUA) by similar ligand exchange methods (named as InP/ZnS-MPA and InP/ZnS-MUA QDs, details in Supplementary Method 7), which are two widely used organic thiols for ligand exchange. Interestingly, the hydrogen evolution efficiency with these two QD types is considerably below that of InP/ZnS-S QDs (Fig. 5a). Similar phenomena have been observed in field effect transistors and in solar cells based on QDs with inorganic ligands over the past years^30,31,57,58^. Recent work also shows the inhibitory effect of thiol ligands on CdS QDs for photocatalysis^59^, and exceptional hydrogen evolution efficiency was achieved based on CdSe/CdS QDs with S^2−^ ligands^32^. So here we attribute the superior hydrogen evolution efficiency with InP/ZnS-S QDs to the replacement of conventional organic ligands by inorganic sulfide ligands.

Transient photocurrent measurements (Fig. 5b) show that InP/ZnS-OLA QDs barely respond to light. In contrast, InP/ZnS-MPA QDs and InP/ZnS-S QDs displayed a quick light response. Especially InP/ZnS-S QDs exhibited a higher current density than InP/ZnS-MPA QDs, i.e., InP/ZnS-S QDs exhibit favorable interaction with their reaction system. Additionally, electrochemical impedance spectroscopy (EIS) data show that FTO electrodes covered with InP/ZnS-S QDs exhibit the lowest resistance under light illumination among the test series (Fig. 5c). Fitting results indicated that R_ct_ was 2.3 × 10^4^, 7.8 × 10^4^, 1.4 × 10^6^, and 6.2 × 10^5^ Ω for FTO covered with InP/ZnS-S, -MPA, -OLA QDs and blank FTO, respectively (see Supplementary Fig. 22 and Supplementary Table 8). Note that the R_ct_ of InP/ZnS-OLA QDs even exceeds the blank value, thereby indicating that organic ligands inhibit charge transportation. Alternatively, InP/ZnS-S QDs may display more active interfaces compared to InP/ZnS-MPA QDs. Compared to organic ligands, such as OLA and MPA, the single anion S^2−^ can far more easily circumvent the physical and electrical barriers arising from the large volume and insulator carbon chains of organic ligands^58,60,61^. This may facilitate the migration of charge carriers from the QDs towards other components of the reaction systems, in particular H_2_A or Ni^2+^. Additionally, this may be the reason why InP/ZnS-MPA QDs are much better photosensitizers than InP/ZnS-MUA QDs, given that the carbon chain of MPA (3) is shorter than that of MUA (11).

After confirming the superior activity of sulfide ligands over organic thiol ligands in the present system for hydrogen evolution, we furthermore synthesized InP/ZnS QDs capped with Cl^−^ and PO_4_^3−^ (denoted as InP/ZnS-Cl and InP/ZnS-PO_4_ QDs, details in Supplementary Method 8) to explore possible differences between InP/ZnS-S QDs and those with other inorganic ligands. Interestingly, the hydrogen evolution efficiency in the presence of these QDs was also much lower than that of InP/ZnS-S QDs (Fig. 5d and Supplementary Fig. 23 for InP QDs). In combination with the above results, we tentatively attribute this to the effective hole transfer from the intrinsic QDs to sulfide, which is more difficult for chloride and phosphide due to their relatively positive oxidation potential.

Electron paramagnetic resonance (EPR) spectroscopy was then performed to obtain further information on the different all-inorganic QD types. As shown in Fig. 5e and Supplementary Fig. 24, no obvious signals could be observed for all QDs in the dark. With light on, still no obvious signal was recorded for InP/ZnS-Cl and -PO_4_ QDs. However, for InP/ZnS-S QDs, a signal around a *g* factor of 2.006 immediately emerges after light irradiation. The absence of this signal in the dark indicates that it is highly related to the photogenerated exciton, rather than to the intrinsic structure defects or surface states of the QDs^62^. Careful examination further shows that it does not arise from photogenerated holes in InP QDs (*g* = 2.001)^62,63^. Instead, it is probably due to electrons in the conduction bands of InP QDs (g = 2.006) according to the relevant literature^62,63^. This hypothesis was further confirmed by control experiments: after the introduction of H_2_A into the system to deplete the photogenerated holes, this signal became much more evident (Supplementary Fig. 24). As all the QDs in our test are derivatives of InP/ZnS-OLA QDs, their interior exciton processes should be similar. We therefore believe that this electron related signal in our system is directly related to the hole capture ability of the surface ligands. Accordingly, we conclude that holes in InP/ZnS-S QDs effectively transfer from the interior to surface S^2−^, which in return significantly increases the stability of electrons in the conducting band and renders them detectable in the EPR spectra at room temperature. As a contrast, the hole transfer from QDs to Cl^−^ or PO_4_^3−^ is limited and the relevant signals are not obvious (Fig. 5e).

Moreover, surface photovoltage spectra (SPV) of InP/ZnS-S, -OLA, -MPA, and -Cl QDs were recorded to obtain more information about different charge separation and transportation characteristics of S^2−^ capped QDs compared to those equipped with other typical organic and inorganic ligands. No photovoltage existed beyond 600 nm for QDs, and this is in accordance with the absorption spectrum of QDs, meaning that no sub-band gap excitation from the trapped state contributes to the photogenerated charge transfer process^64^. The corresponding photovoltage generated by -S QDs is obviously much larger than that of -OLA, -Cl, and -MPA QDs (Fig. 5f), confirming the efficient charge separation of the sulfide capped particles once again^65^. The positive response means that positive charge accumulated at the interface under irradiation^66^, further demonstrating that sulfide ligands extract holes to the surface of InP/ZnS-S QDs, in line with the above TA and EPR experiments. Besides, strong interdot coupling and band-like charge transport may exist in QDs capped with inorganic ligands, such as S^2−^ or Cl^−^^30,67^, which could contribute to their higher charge mobility and resulting higher photovoltage in contrast to MPA and OLA capped QDs.

Furthermore, density functional theory (DFT) calculations (Supplementary Method 1, Supplementary Figs. 25–27, and Supplementary Note 3) also indicate that introduction of S centers on the surface of the QDs is beneficial for charge transfer, which is consistent with the above experiments and analyses. Moreover, the nature of S^2−^ ligands is different from the intrinsic defects on InP QDs, and the latter can be suppressed by the introduction of ZnS.

## Discussion

As the surface S^2−^ ligands may be regarded as a kind of surface trap, which is indicated by the decrease of the QD luminescence, we further analyze the different role of S^2−^ ligands compared to the ordinary intrinsic defects of QDs according to the above experimental measurements and theoretical calculations (Supplementary Fig. 28). The ordinary intrinsic defects on QDs are often disordered, and they always act as recombination centers for holes and electrons, which both lowers the band-edge emission of QDs and reduces the charge separation efficiency^68^. In our work, the introduction of ZnS on InP QDs can be considered as a suitable strategy to decrease the amount of intrinsic defects which are detrimental for photocatalysis with QDs. The effectiveness of this approach is proven by the higher PLQY and hydrogen evolution efficiency after ZnS layer growth. Alternatively, the introduction of S^2−^ ligands can be regarded as an exterior trap^69^. Although the surface S^2−^ ligands decrease the emission of QDs in the present case, they improve the charge separation efficiency of the system because of their hole capture ability. That is, after light excitation of QDs, the holes can transfer quickly to the S^2−^ ligands, which is beneficial for electron/hole separation. Moreover, the small volume of S^2−^ in comparison to the traditional organic ligands lowers the barrier for further hole transfer from S^2−^ to H_2_A.

In summary, we have successfully introduced inorganic S^2−^ capped InP and InP/ZnS QDs as efficient photosensitizers for hydrogen evolution. InP/ZnS QDs reach TON values up to 128,000 per QDs and an internal quantum yield of 31% (525 nm). This demonstrates the remarkable potential of S^2−^ capped InP-based QDs for photocatalytic applications. Moreover, using a series of spectroscopic and photoelectrochemical characterizations, we demonstrate that inorganic sulfide ligands on QDs are beneficial for the photocatalytic hydrogen evolution. In comparison with conventional organic ligands and other inorganic ligands, sulfide ions on the surface can extract the holes from the interior of QDs rapidly, followed by their reaction with H_2_A via low physical and electronic barriers. This leads to efficient charge separation and notably enhances the photocatalytic hydrogen evolution performance. Key perspectives for further progress are the development of other InP-based heterostructures, and the application of inorganic sulfide capped InP QDs for a wider spectrum of photocatalytic reactions.

## Methods

### Synthesis of 525 nm InP and InP/ZnS QDs

In total 111 mg (0.5 mmol) of indium(III) chloride as indium precursor, 204 mg (1.5 mmol) of zinc(II) chloride and 160 mg (0.5 mmol) zinc(II) iodide as zinc precursors were mixed in 5.0 mL (15 mmol) of oleylamine. Here Zn precursors were added to assist in controlling the size distribution of InP QDs^28^. The reaction mixture was then evacuated by Schlenk techniques and kept under vacuum at 120 °C for 1 h. Afterwards, the system was heated to 180 °C under inert atmosphere. 0.5 mL (1.8 mmol) of tris(diethylamino)phosphine (phosphorous: indium ratio = 3.6: 1) was then quickly injected into the mixture. The system was kept at 180 °C for 30 min to drive the growth of InP QDs to completion and then cooled to room temperature.

For InP/ZnS QDs, after the above system was kept at 180 °C for 30 min, it was further heated to 260 °C instead of cooling down, and 1 mL of trioctylphosphine (TOP)-S (2 M) solution was slowly added with a rate of 0.2 mL min^−1^ (TOP-S solution was made by dissolving 0.128 g of sulfur in 2 mL of TOP by heating or ultrasound under inert atmosphere). Time measurement for ZnS layer growth was started after the whole TOP-S solution had been added, and the reaction was stopped after different times. Finally, the system was cooled down to room temperature.

After the above crude QD solution was cooled down to room temperature, about 10 mL of ethanol (EtOH) were added for the precipitation of QDs. After centrifugation, the supernatant was discarded. The precipitated QDs were further purified by dissolution in 10 mL of hexane (HEX) and subsequent precipitation in 15 mL of ethanol. Finally, the QDs were again dissolved in 20 mL of HEX and centrifuged to remove the insoluble impurities. The prepared QDs could be well kept in solution at 2–6 °C.

### Synthesis of QDs with different excitonic absorption peaks

To synthesize InP and InP/ZnS QDs with excitonic absorption peaks located at other wavelengths, different ratios of zinc(II) chloride and zinc(II) iodide precursors were added. The remaining processes were performed as described above. In summary: when 2 mmol of zinc(II) chloride was used, QDs with an absorption peak around 570 nm were obtained; when 1 mmol of zinc(II) chloride and 1 mmol of zinc(II) iodide were used, QDs with an absorption peak around 480 nm were obtained; when 0.5 mmol of zinc(II) chloride and 1.5 mmol of zinc(II) iodide were used, QDs with an absorption peak around 435 nm were obtained. For QDs with short absorption peak, the purification process is somewhat more demanding (see Supplementary Note 1). For the sake of clarity, we refer to InP QDs with excitonic absorption peak at X nm as InP QDs (X nm) and to InP/ZnS with ZnS growth time of Y min as InP/ZnS QDs (Y min).

### S^2−^ ligand exchange for InP and InP/ZnS QDs

Solutions for ligand exchange with S^2−^ were prepared by dissolving Na_2_S·9H_2_O in highly polar solvents, namely formamide (FA) or N-methylformamide (NMF). Ligand exchange with S^2−^ can be carried out directly under ambient conditions and usually proceeded within 5 min to 1 h, depending on the kind of polar solvent, concentrations of QDs and S^2−^, stirring speed and temperature. No obvious difference was observed when the ligand exchange was performed in air or under inert atmosphere. In a typical process, 1 mL of InP or InP/ZnS QDs (about 3–5 mg mL^−1^) in HEX were mixed with 1 mL of Na_2_S·9H_2_O (0.05 M) solution dissolved in NMF. The mixture was kept stirring at room temperature until a total phase transfer for QDs from HEX to NMF was achieved. This transfer process can be easily monitored by the color change of the HEX (red to colorless) and NMF (colorless to red) phases. The HEX phase was then discarded and the NMF phase was further washed with HEX twice to remove any remaining nonpolar organic species. The washed NMF phase was precipitated with 3 mL acetone and purified with 0.5 mL FA and 1 mL acetone. Finally, the precipitate was redissolved in water by ultrasound. Afterwards, the solution was centrifuged to remove any insoluble QDs and impurities, and the obtained colloidal aqueous solution of QDs was stored at 2–6 °C.

### Photocatalytic hydrogen evolution tests

Typical hydrogen evolution experiments were performed with 6 mL of H_2_O solution containing 1.6 μM QDs, 0.035 mM NiCl_2_·6H_2_O, and 0.2 M H_2_A/NaHA in a Pyrex tube at pH 4.5. The pH of the mixed solution can be easily adjusted by changing the ratio of H_2_A to NaHA. After deoxygenation with argon for 20 min, the system was irradiated with a home-made LED reactor with 4 pcs of 525 nm bulbs (Tao Yuan, China; 4 × 1 W) under constant stirring and circular water cooling. Other light sources such as 4 pcs of 465 nm LED (Tao Yuan, China; 4 × 1 W) and AM 1.5 simulator (LOT-Oriel, LS0306) were also used in the photocatalytic experiments. The photogenerated H_2_ was quantified by GC (Varian CP-3800) using argon as the carrier gas with a molecular sieve column (5 Å) and a thermal conductivity detector. To ensure reproducibility, two or more independent measurements were performed. As a data treatment, the standard deviation (showed as error bar) was increased to 5% of the average value if it was below this threshold^15^.

In addition, we also used an on-line hydrogen evolution and detection system developed in house. The system was irradiated by one LED bulb with 453 nm wavelength of tunable intensity. Gas chromatograms were recorded using an automated Bruker GC 456 gas chromatograph with argon as the carrier gas and a 3 m × 2 mm packed molecular sieve 13 × 80–100 column. The column and reference gas flow (Ar) were set to 20 mL min^−1^. The oven was operated isothermally at 100 °C. An argon flow of 6 mL min^−1^ (adjusted with an onboard EFC device and referenced with a F-200CV-002 from Bronkhorst) was passed through the reaction mixture and into the GC, where 1 mL gas samples were automatically injected in defined time intervals (5 min) using a 6-Port-2-position Valve from Vici. The gases were detected using a thermal conductivity detector operated at 150 °C (retention time is about 1 min for H_2_). Data were then analyzed with the software Origin Pro.

## Electronic supplementary material

Supplementary Information

## Data Availability

The data that support the findings of this study are available from the corresponding authors upon request.
