# Supplementary material for: Efficient photocatalytic hydrogen evolution with ligand engineered all-inorganic InP and InP/ZnS colloidal quantum dots
Source: Nat Commun. 2018 Oct 1;9:4009. doi: 10.1038/s41467-018-06294-y (PMC6167351; doi:10.1038/s41467-018-06294-y)
Supplement: Supplementary file 1 — Supplementary Information [file 41467_2018_6294_MOESM1_ESM.pdf]

Supplementary Information

Efficient Photocatalytic Hydrogen Evolution with Ligand Engineered All-Inorganic InP and InP/ZnS Colloidal Quantum Dots

Yu *et al.*

Supplementary Figures.

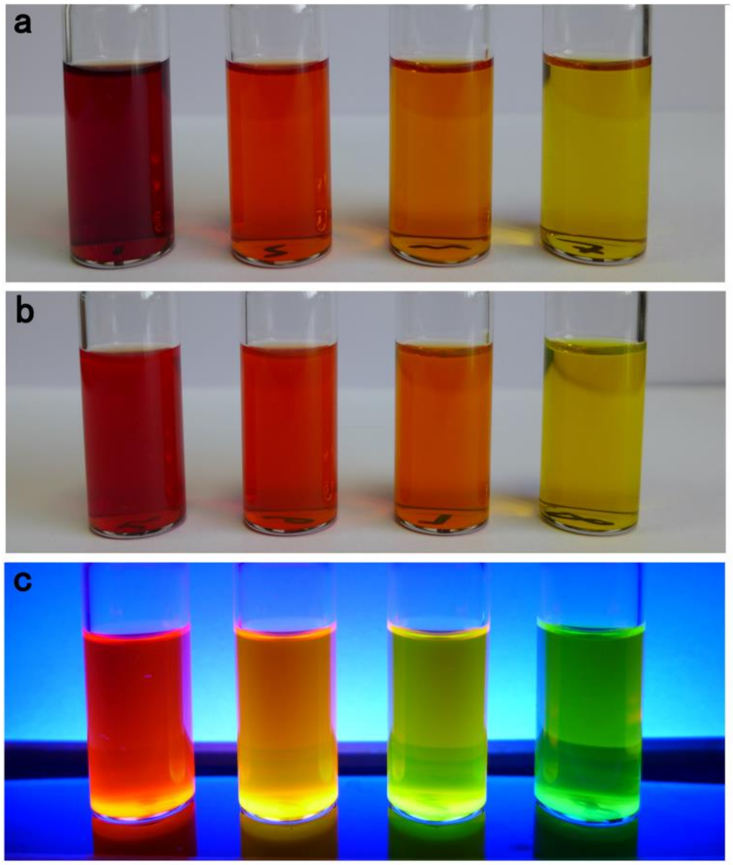

Supplementary Fig. 1. Photographs of as-synthesized QDs with different absorption peaks. **a.** InP; **b.** InP/ZnS QDs (15 min) and **c.** InP/ZnS QDs (15 min) under ultraviolet irradiation. From left to right: QDs with absorption peaks at 570 nm, 525 nm, 480 nm and 435 nm, respectively.

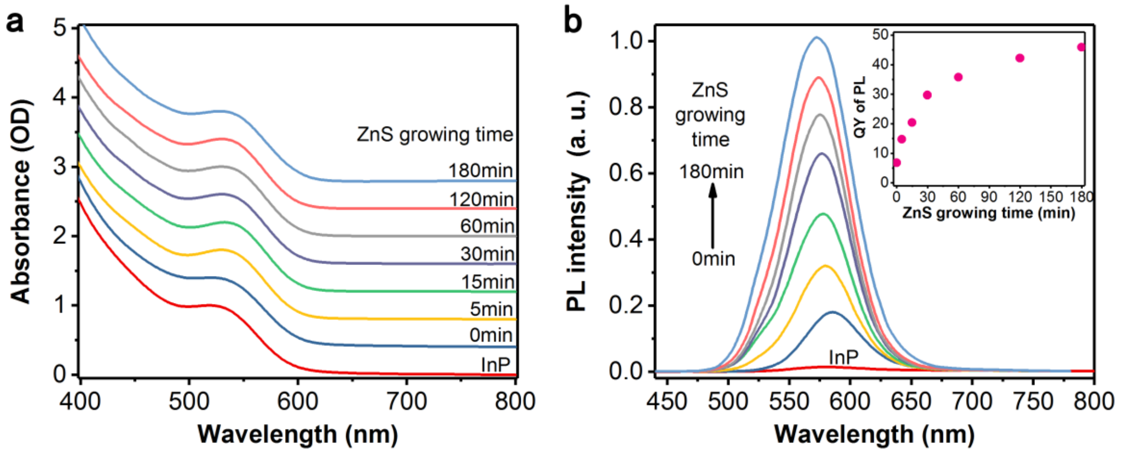

Supplementary Fig. 2. Absorption spectra and PL spectra of InP QDs with different growth times of ZnS. **a.** Absorption spectra; **b.** PL spectra recorded for different growth times of ZnS on InP QDs (525 nm); inset: corresponding photoluminescence quantum yield (PLQY, %) changes of InP/ZnS QDs.

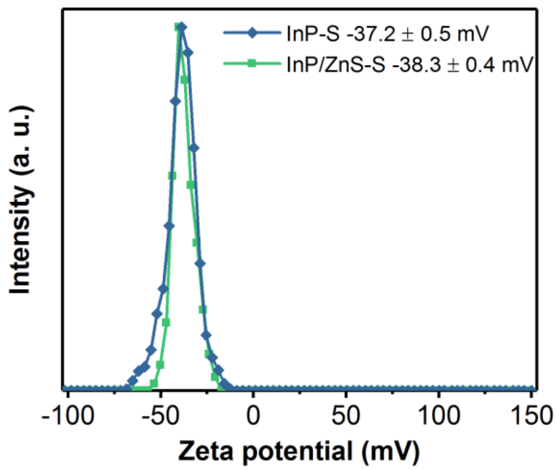

Supplementary Fig. 3. Zeta potential of S^{2-} capped InP QDs (525 nm) and InP/ZnS QDs (525 nm, 15 min) in water.

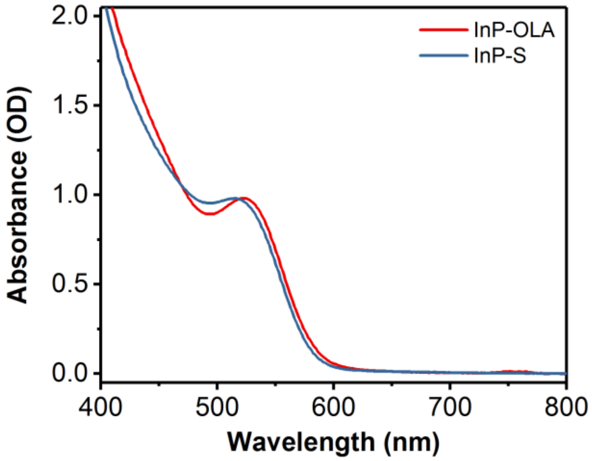

Supplementary Fig. 4. Absorption spectra before and after ligand exchange for InP QDs (525 nm) in hexane and water.

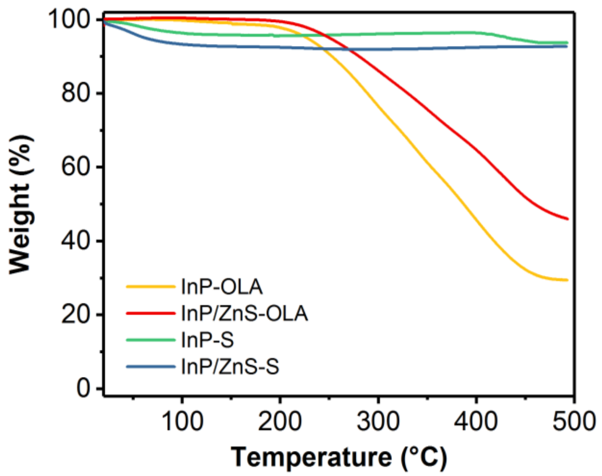

Supplementary Fig. 5. Thermogravimetric scans for InP (525 nm) and InP/ZnS (525 nm, 15 min) QDs capped with different organic ligands and sulfide anions.

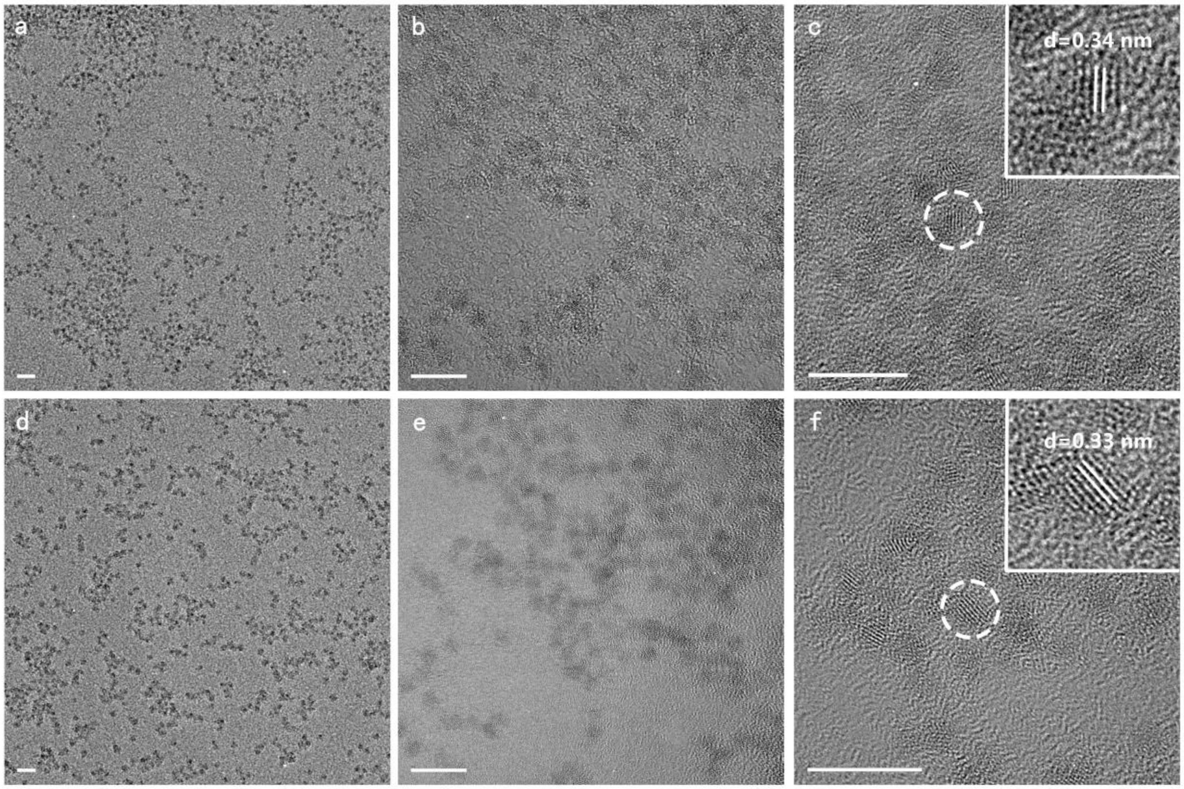

Supplementary Fig. 6. TEM images of QDs with OLA ligands. **a-c.** InP-OLA QDs (525 nm); **d-f.** InP/ZnS-OLA QDs (525 nm, 15 min); scale bar: 10 nm.

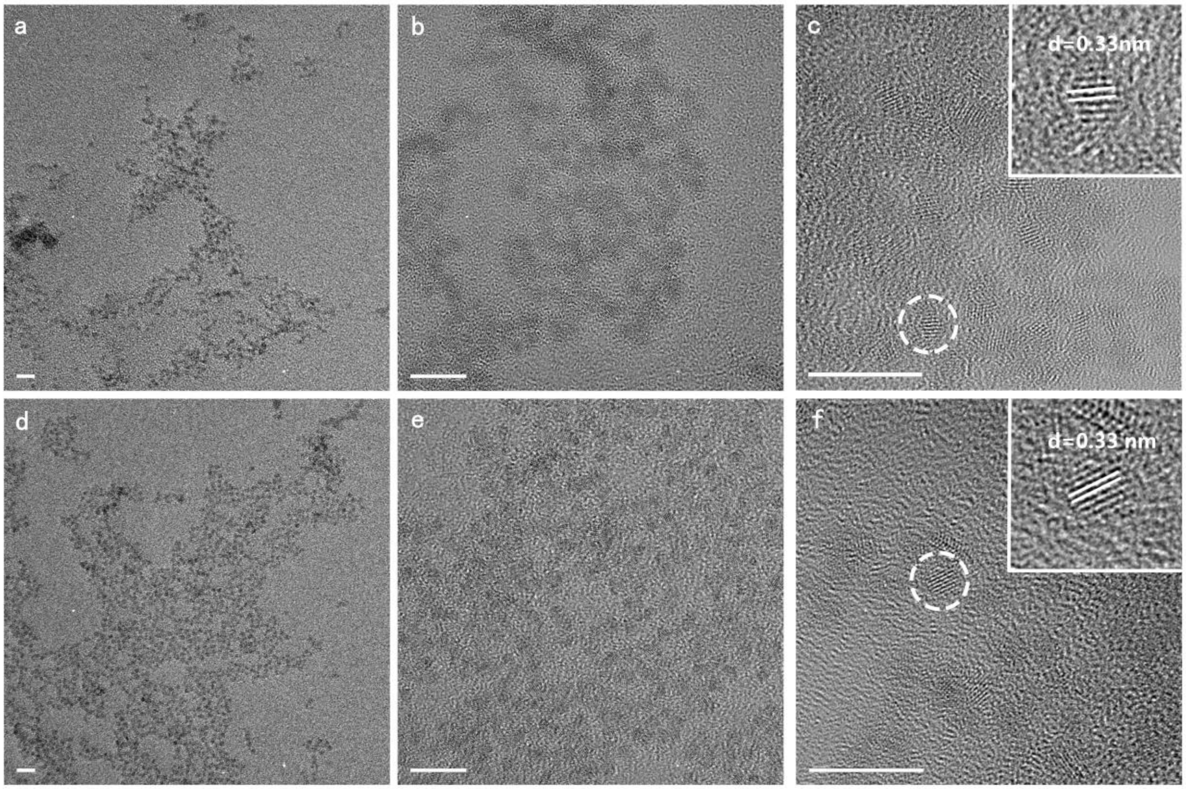

Supplementary Fig. 7. TEM images of QDs with S^{2-} ligands. **a-c.** InP-S QDs (525 nm); **d-f.** InP/ZnS-S QDs (525 nm, 15 min); scale bar: 10 nm.

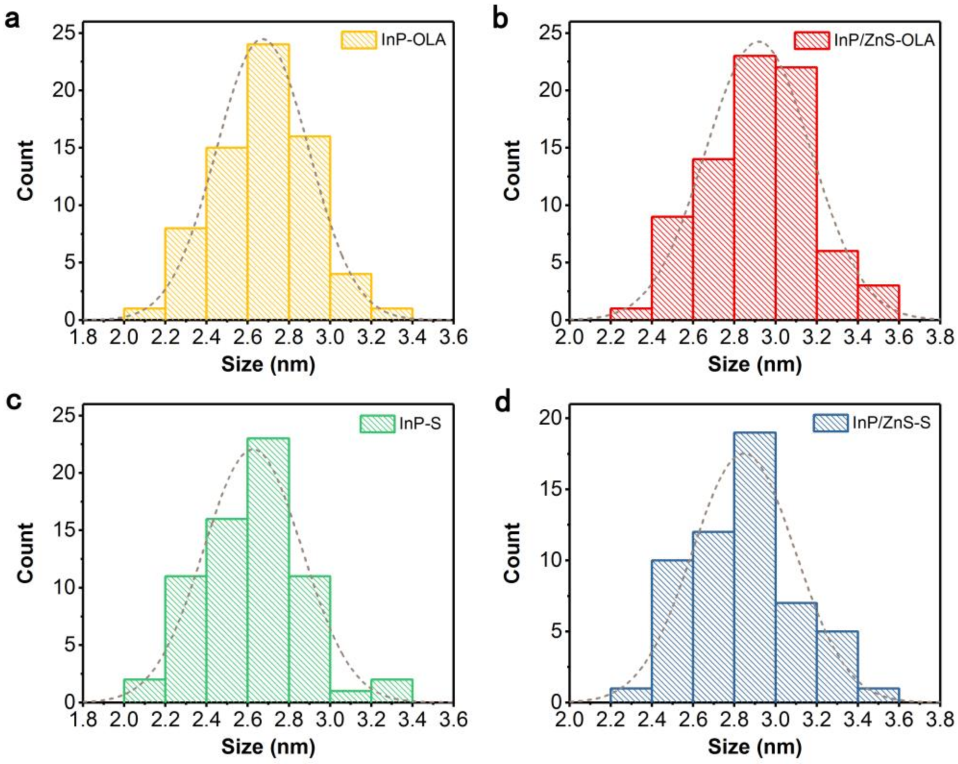

Supplementary Fig. 8. Statistical analysis of the size distribution of QDs from TEM images. **a.** InP-OLA QDs (525 nm); **b.** InP/ZnS-OLA QDs (525 nm, 15 min); **c.** InP-S QDs (525 nm) and **d.** InP/ZnS-S QDs (525 nm, 15 min).

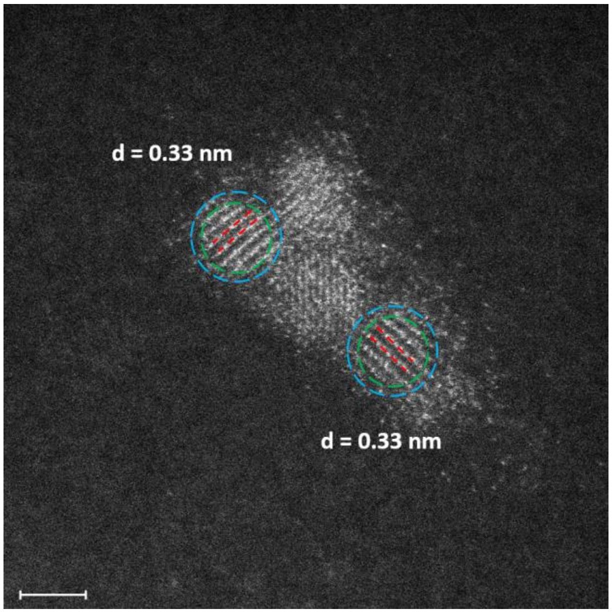

Supplementary Fig. 9. HAADF-STEM image of InP/ZnS-S QDs (525 nm, 15 min); scale bar: 2 nm.

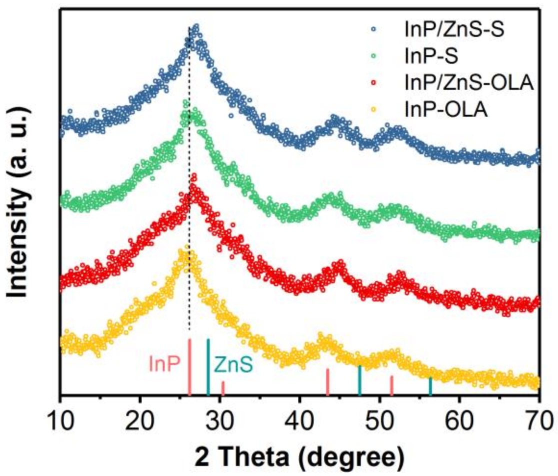

Supplementary Fig. 10. PXRD patterns of InP (525 nm) and InP/ZnS (525 nm, 15 min) before (QDs-OLA) and after ligand exchange (QDs-S).

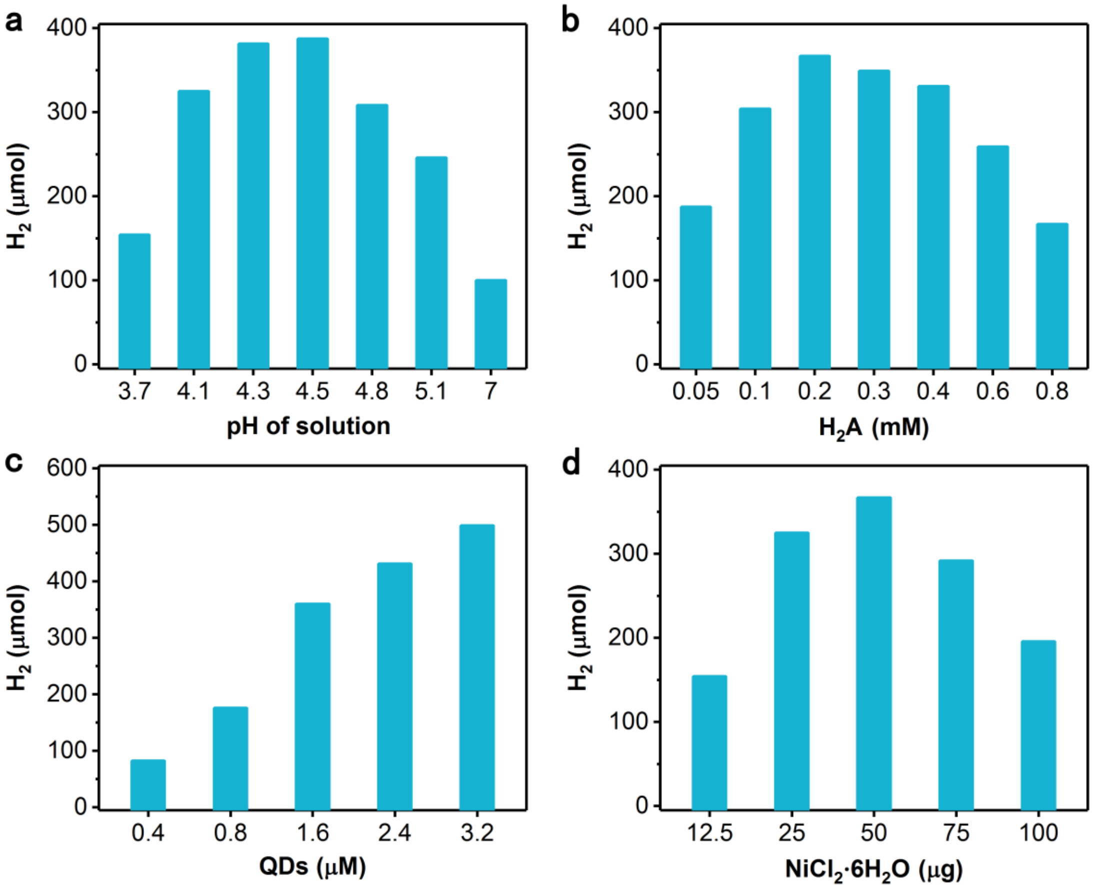

Supplementary Fig. 11. Optimization of photocatalytic hydrogen evolution with InP/ZnS-S QDs (525 nm, 15 min). *Reaction conditions*: **a.** 6 mL solution containing 0.2 M H_2A/NaHA, 1.6 μM QDs and 0.05 mg $NiCl_2 \cdot 6H_2O$ at different pH values; **b.** 6 mL solution containing 1.6 μM QDs, 0.05 mg $NiCl_2 \cdot 6H_2O$ and different concentrations of H_2A/NaHA at pH 4.5; **c.** 6 mL solution containing 0.2 M H_2A/NaHA, 0.05 mg $NiCl_2 \cdot 6H_2O$ and different amounts of QDs at pH 4.5; **d.** 6 mL solution containing 0.2 M H_2A/NaHA, 1.6 μM QDs and different amounts of $NiCl_2 \cdot 6H_2O$ at pH 4.5. Light source: 525 nm LED light irradiation (4 × 1 W); illumination time: 2 h.

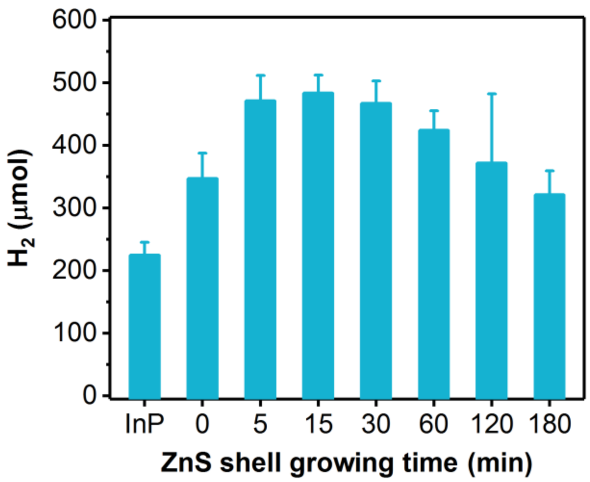

Supplementary Fig. 12. Photocatalytic hydrogen evolution with InP/ZnS-S QDs (525 nm) with different ZnS growth times. *Reaction conditions*: 6 mL solution containing 0.2 M H_2A, 1.6 μM QDs, and 0.035 mM $NiCl_2$ at pH 4.5. Light source: 525 nm LED light irradiation (4 × 1 W); illumination time: 3 h. Error bars were estimated based on the standard deviation according to two or more independent experiments.

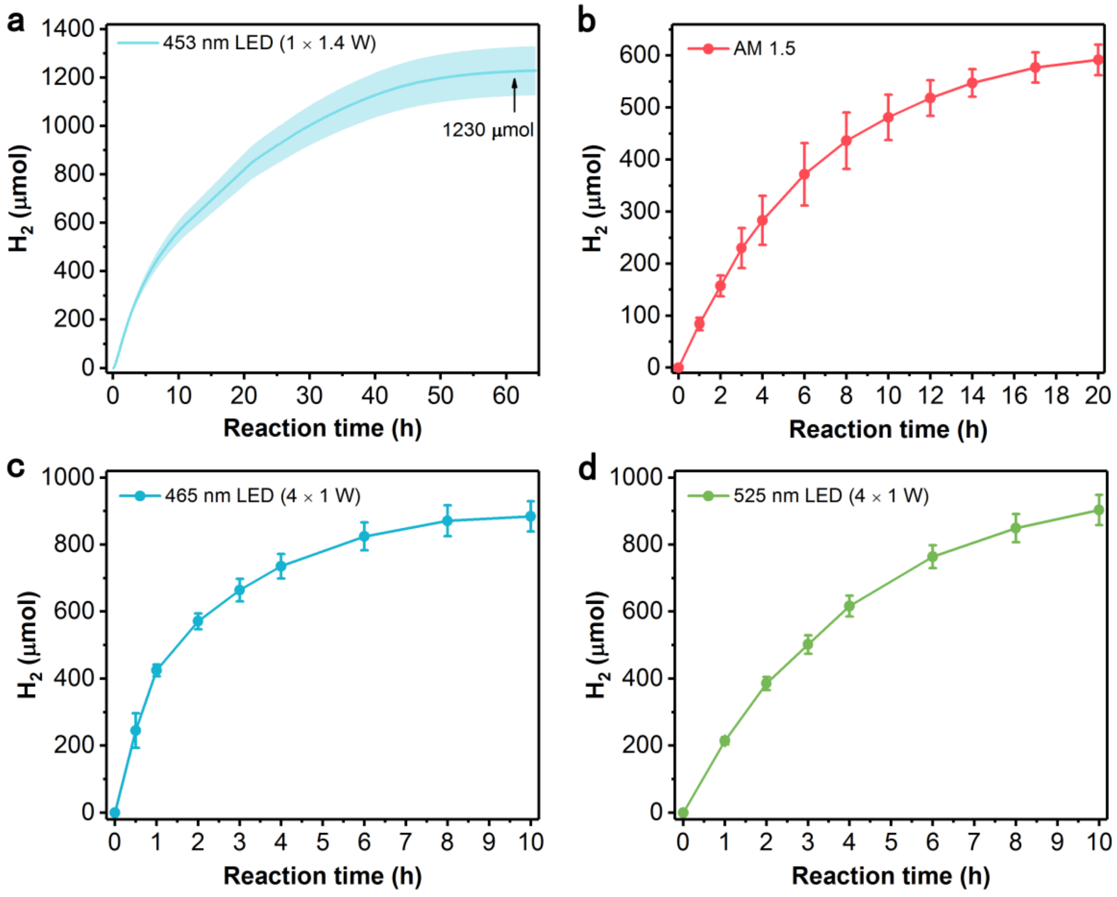

Supplementary Fig. 13. Photocatalytic hydrogen evolution with InP/ZnS-S QDs (525 nm, 15 min) with other light sources. Photocatalytic hydrogen evolution for systems containing 9.6 nmol of InP/ZnS QDs, 0.21 μmol $NiCl_2$ and 0.2 M H_2A (pH 4.5) under illumination from different light sources: **a.** LED light source (453 nm, 1 × 1.4 W, shadow area: error bar); **b.** simulated sunlight (AM 1.5, 100 mW cm^{-2}); **c.** LED light source (465 nm, 4 × 1 W), and **d.** LED light source (525 nm, 4 × 1 W). Hydrogen evolution for **a** was detected with an on-line system (10 mL solution) while the other measurements were conducted off-line (6 mL solution). Error bars were estimated based on the standard deviation according to three independent experiments.

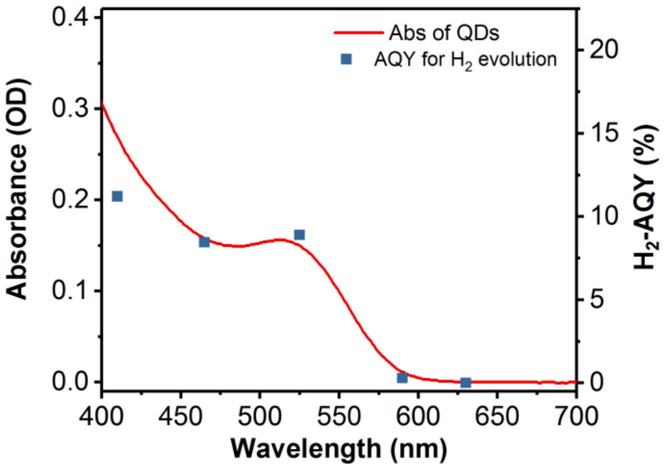

Supplementary Fig. 14. Comparison of the absorption spectrum of InP/ZnS-S QDs (525 nm, 15 min) with AQY values for photocatalytic hydrogen evolution.

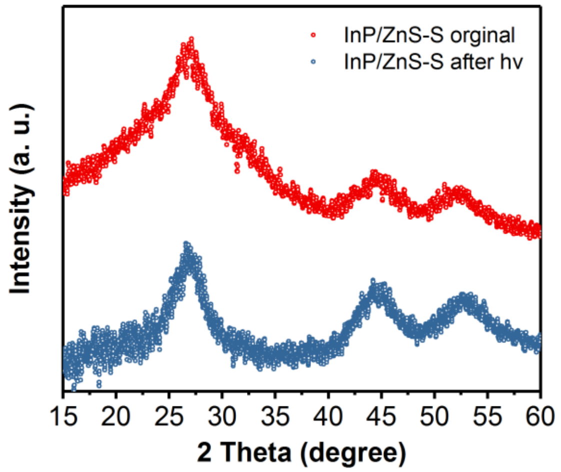

Supplementary Fig. 15. PXRD patterns of InP/ZnS-S QDs before and after illumination. *Reaction conditions:* LED light source (453 nm, 3.4 W) over 5.5 h, which leads to generation of 14.0 mL H_2.

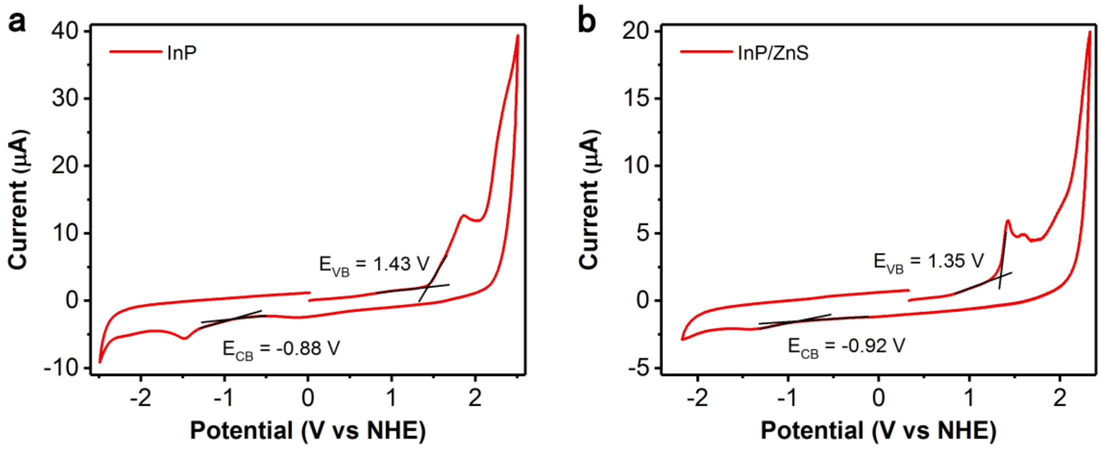

Supplementary Fig. 16. Band gap calculation of InP/ZnS QDs from CV measurements. Cyclic voltammogram of **a.** InP (525 nm) and **b.** InP/ZnS QD (525 nm, 15 min) in acetonitrile. Electrolyte: tetrabutylammonium hexafluorophosphate ($TBAPF_6$, 0.1 M); scan rate: 50 mV s^{-1}. The redox potential was calibrated with the ferrocene/ferrocenium redox couple (E_{Fc/Fc^+} = 0.64 vs. NHE).

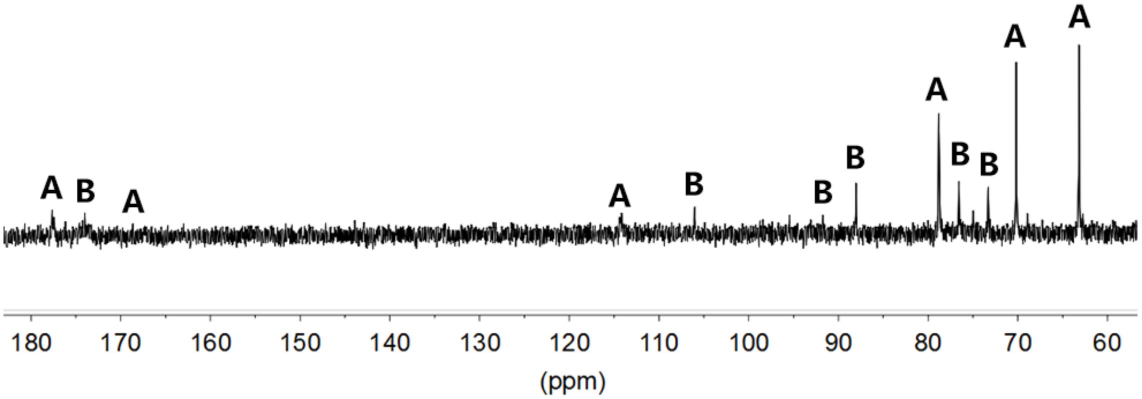

Supplementary Fig. 17. Detection of dehydroascorbic acid (DHA) by ^{13}C NMR after photocatalytic hydrogen evolution. A = peaks associated with ascorbic acid (H_2A), B = dehydroascorbic acid (DHA) hydrate.[1] *Reaction conditions:* 4 h illumination (525 nm, 4 × 1 W) of a photocatalytic solution with InP/ZnS-S QDs, Ni^{2+} and H_2A/NaHA in D_2O.

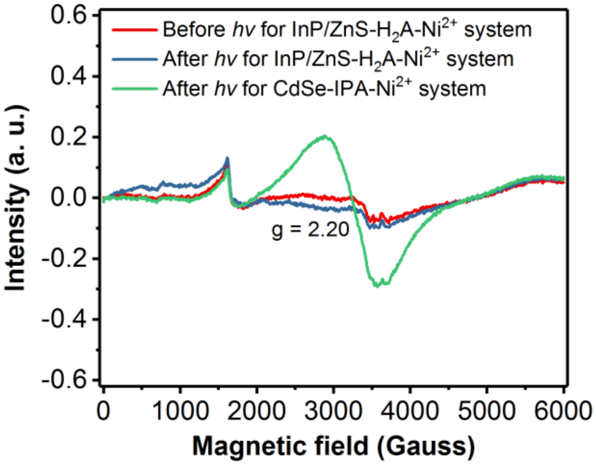

Supplementary Fig. 18. Detection of metallic nickel before and after photocatalytic hydrogen evolution by EPR. If metallic Ni existed, a very wide peak around g = 2.2 would be detected.[2] As a reference, we have also studied another photocatalytic system of CdSe QDs, Ni^{2+} and isopropanol, in which the metallic Ni signal is obvious after illumination.

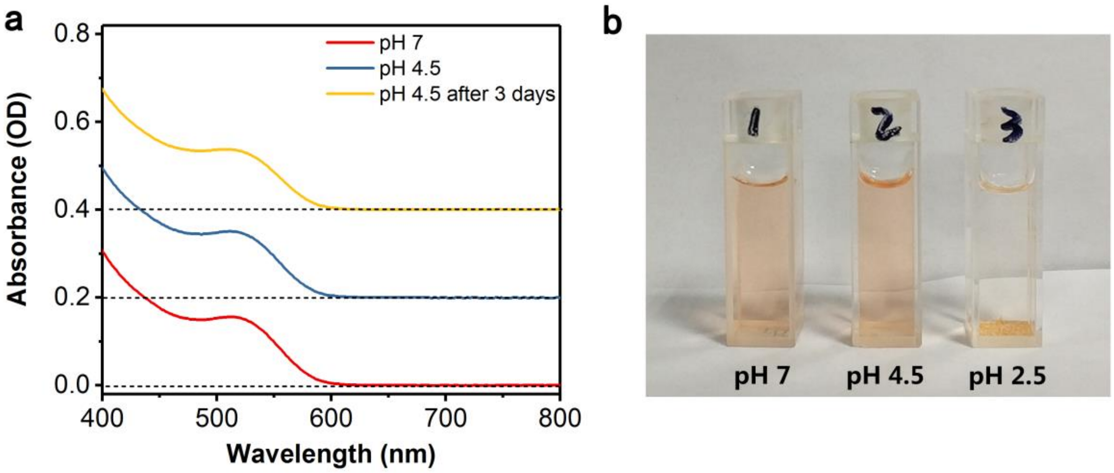

Supplementary Fig. 19. Influence of pH on QDs in photocatalytic media. **a.** absorption spectra of InP/ZnS-S QDs at different pH values; **b.** photograph of InP/ZnS-S QDs at different pH values.

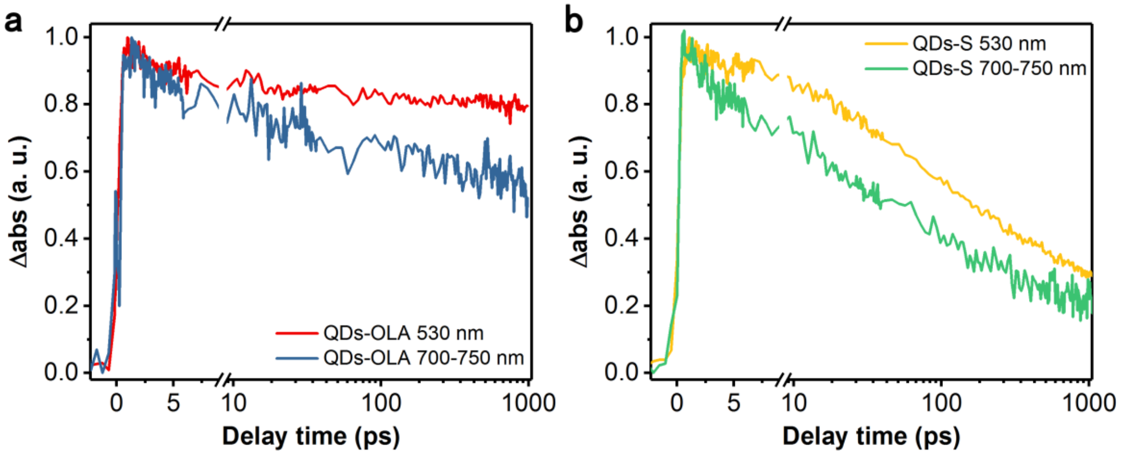

Supplementary Fig. 20. Kinetics comparison for XB (530 nm) and PA (700-750 nm) signals of QDs. **a.** InP/ZnS-OLA QDs; **b.** InP/ZnS-S QDs.

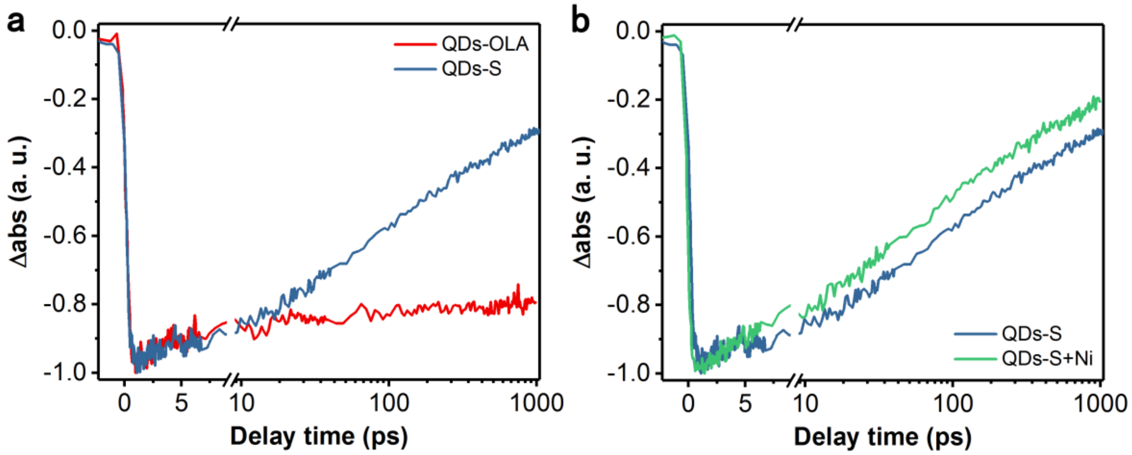

Supplementary Fig. 21. Kinetic analysis for XB signal (530 nm) of QDs. **a.** InP/ZnS-OLA and InP/ZnS-S QDs; **b.** InP/ZnS-S QDs with and without introduction of Ni^{2+} into the solution.

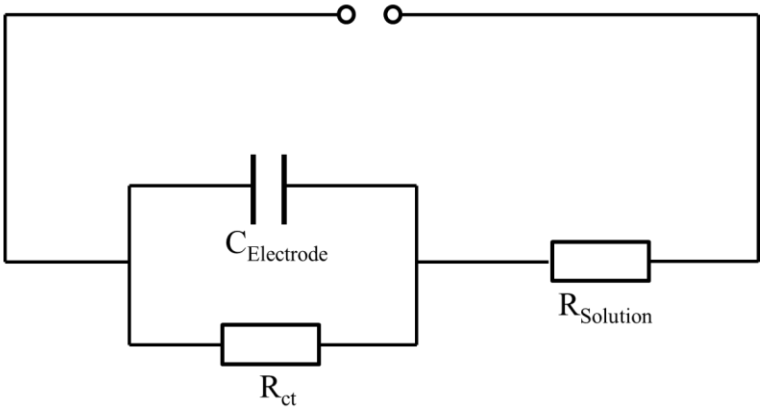

Supplementary Fig. 22. Simulated circuit for fitting of EIS data.

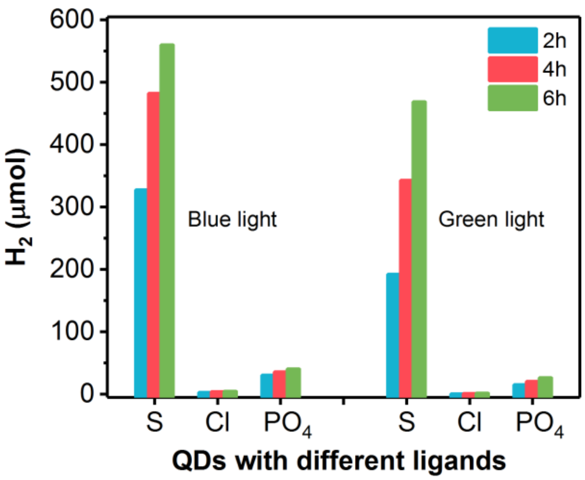

Supplementary Fig. 23. Hydrogen evolution for InP QDs capped with different inorganic ligands. *Reaction conditions:* 1.6 μM QDs, 0.035 mM Ni^{2+} in 6 mL of 0.2 M H_2A (pH 4.5) were illuminated with a LED light source (465 nm for blue light and 525 nm for green light, 4 × 1 W).

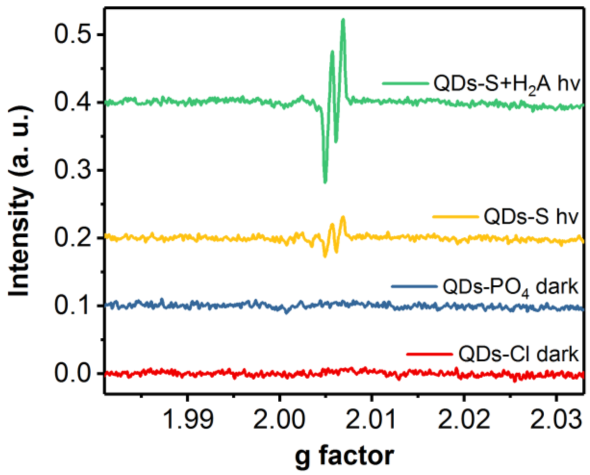

Supplementary Fig. 24. EPR spectra for InP/ZnS QDs with different inorganic ligands. Green and yellow lines represent comparisons of InP/ZnS-S QDs with and without the presence of H_2A, respectively, under light illumination. Blue and red lines represent QDs with PO_4^{3-} or Cl^- in the dark.

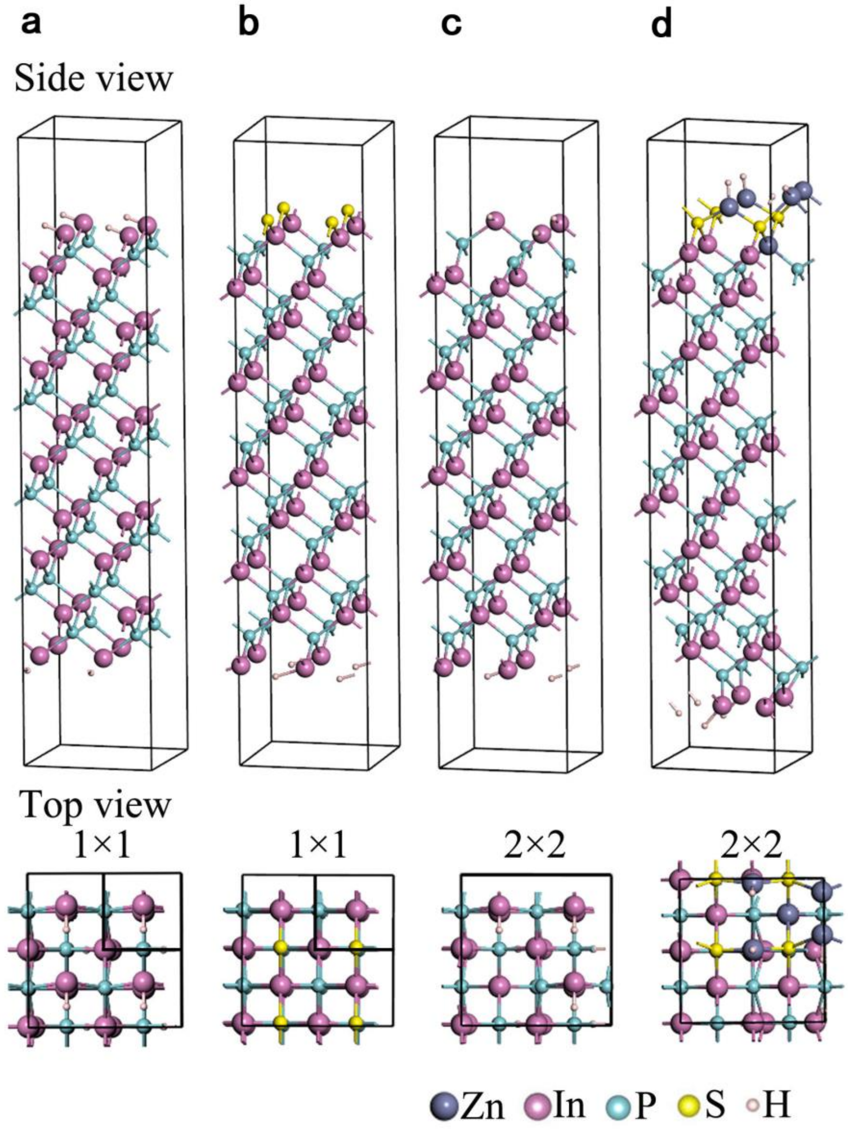

Supplementary Fig. 25. Optimized structures for DFT calculations. **a.** InP-H (InP (001) passivated by pseudo-hydrogen atoms); **b.** InP-S; **c.** InP; **d.** InP/ZnS.

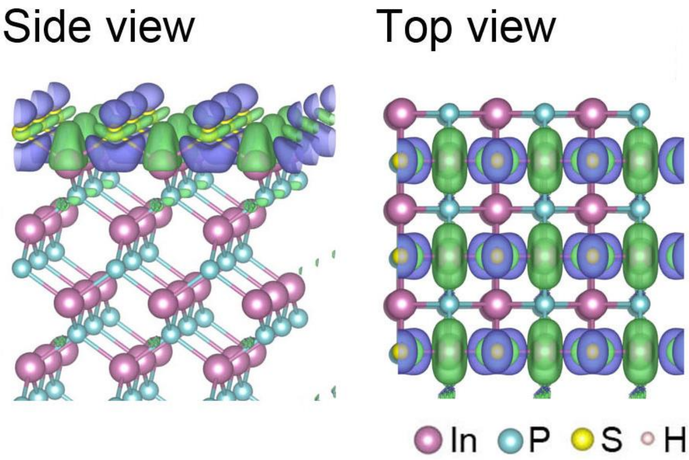

Supplementary Fig. 26. Charge density difference map of InP-S. Charge accumulation: green region, charge depletion: blue region. The isosurface value is 0.003 e $Å^{-3}$.

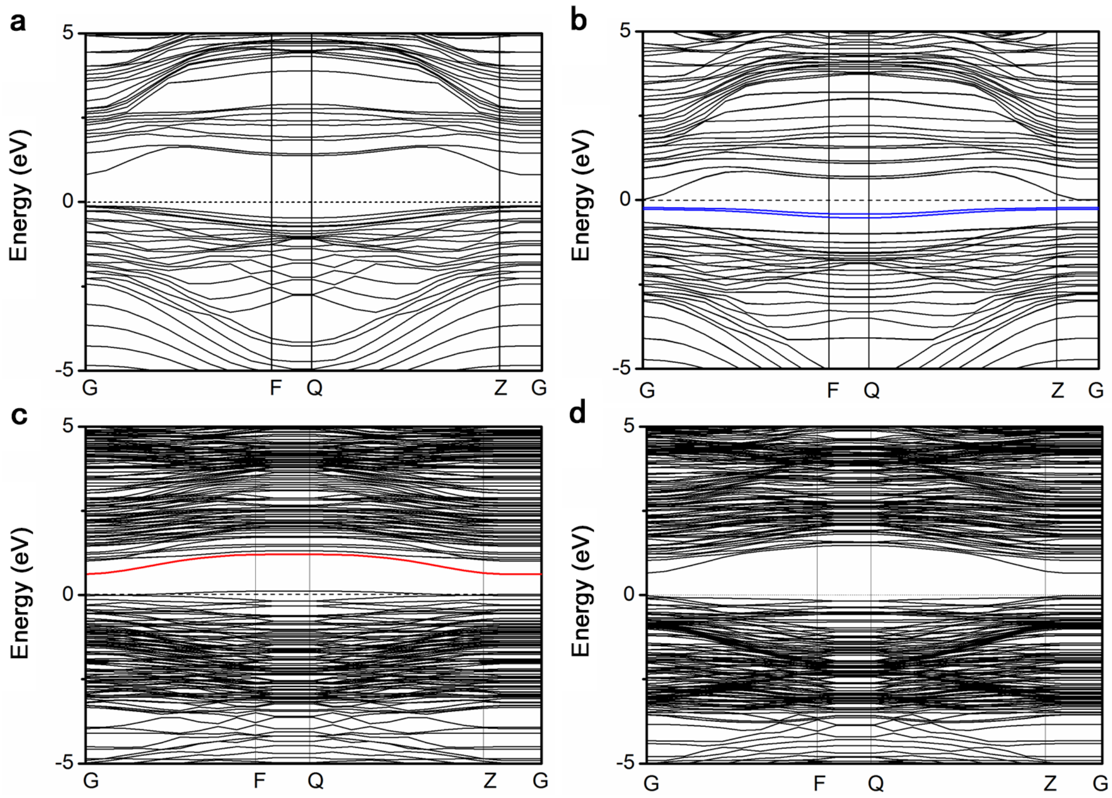

Supplementary Fig. 27. Band structures of InP with different surface states. **a.** InP-H; **b.** InP-S; **c.** InP; **d.** InP/ZnS. Blue and red lines represent extrinsic defects after introduction of S^{2-} and intrinsic defects of InP due to the incomplete surface passivation, respectively.

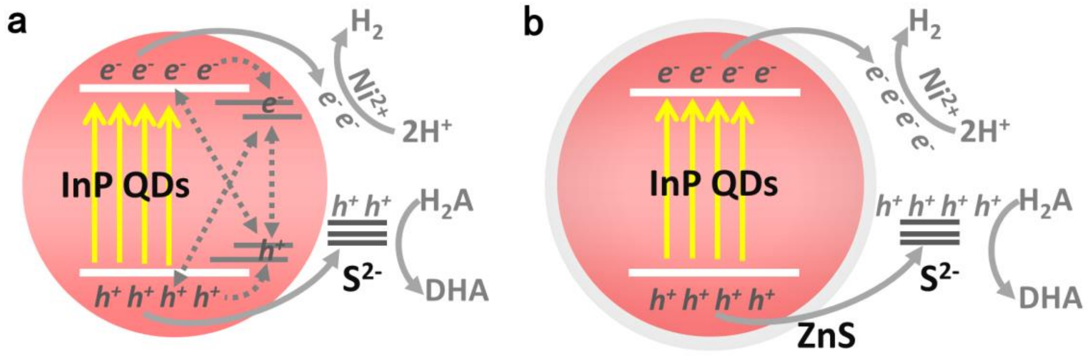

Supplementary Fig. 28. Schematic representation of InP-S and InP/ZnS-S QDs with different defect states. Yellow arrows indicate the photoexcitation processes. Solid grey lines indicate efficacious charge transfer processes contributing to ultimate hydrogen evolution, and dotted lines indicate possible processes resulting in charge recombination, which is detrimental for hydrogen evolution. As the intrinsic defects in InP QDs are eliminated by the introduction of ZnS, charge recombination is inhibited and hydrogen production increases.

Supplementary Tables

Supplementary Table 1. ICP-OES results for QDs before and after ligand exchange. InP QDs (525 nm) and InP/ZnS QDs (525 nm, 15 min) powders were treated with aqua regia for dissolution. As Zn precursors were introduced during the synthesis of InP QDs for size control (cf. Methods in main text), a small amount of Zn could be detected for InP QDs.

	In wt%	P wt%	Zn wt%	S wt%	P/In at%	Zn/In at%	S/In at%
**InP-OLA**	42.4	11.3	3.4	/	0.99	0.14	/
**InP-S**	54.8	11.4	4.5	14.6	0.77	0.14	0.95
**InP/ZnS-OLA**	37.6	8.7	6.6	3.7	0.85	0.31	0.35
**InP/ZnS-S**	50.3	10.8	9.1	13.4	0.80	0.31	0.97

Supplementary Table 2. SEM-EDS data for QDs before and after ligand exchange. InP QDs (525 nm) and InP/ZnS QDs (525 nm, 15 min) powders were spread on a conductive resin for measurements.

	**In at%**	**P at%**	**Zn at%**	**S at%**	**P/In at%**	**Zn/In at%**	**S/In at%**
**InP-OLA**	18.2	16.1	2.7	/	0.88	0.15	/
**InP-S**	13.3	9.6	1.6	9.1	0.72	0.12	0.68
**InP/ZnS-OLA**	13.9	12.7	6.3	4.3	0.91	0.45	0.31
**InP/ZnS-S**	18.8	15.4	8.8	14.5	0.82	0.47	0.77

Supplementary Table 3. QY calculation for InP/ZnS-S QDs (525 nm, 15 min) under different conditions. The IQY at 590 nm is not given due to the large error arising from small hydrogen evolution amounts and low light absorbance.

**Light wavelength (nm)**	**Light intensity (mW)**	**Amount of H_2 (μL) in 0.5 h**	**Absorbance (OD)**	**AQY(%)**	**IQY(%)**
410	55 ± 1	424 ± 31	0.264 ± 0.004	11.2	24.5
465	66 ± 1	435 ± 40	0.158 ± 0.002	8.44	27.6
525	29 ± 1	228 ± 17	0.147 ± 0.003	8.89	31.0
590	16 ± 1	18.3 ± 1.2	0.010 ± 0.001	0.29	-
630	23 ± 1	0	-	0	-

Supplementary Table 4. Zeta potential of InP/ZnS-S QDs under different conditions.

Conditions (aqueous solution)	Zeta potential (mV)
QDs pH 7	-36.1 ± 1.8
QDs pH 4.5	-31.2 ± 0.2
QDs pH 4.5 + Ni^{2+}	-26.9 ± 0.8
QDs pH 4.5 + Ni^{2+} + H_2A/NaHA	-22.5 ± 0.6

Supplementary Table 5. Kinetics fitting result for PA signal of InP/ZnS-OLA and InP/ZnS-S QDs. The result corresponds to Figure 3c in the main text. Kinetics at 725 nm (average from 700 nm to 750 nm) were selected for fitting. We chose the minimum number of exponential processes to obtain a satisfactory fitting result.[3]

QDs	τ_1 (ps)	A_1	τ_2 (ps)	A_2	τ_3 (ns)	A_3
**InP/ZnS-OLA**	9.464	28.1%	/	/	3911	71.9%
**InP/ZnS-S**	8.215	37.6%	109.6	32.7%	3146	29.7%

Supplementary Table 6. Kinetic analysis of emission decay for InP/ZnS-S QDs (525 nm, 15 min) in the presence of different amounts of Ni^{2+}. Ni^{2+} was added with a maximum concentration of 0.04 mM, because such conditions are similar to photoreaction conditions (0.035 mM Ni^{2+}). For the measurement of emission spectroscopy, the pH of all solutions was adjusted to 4.5, and the excitation wavelength was 406 nm. The decay traces for the samples were well fitted with a triple-exponential function and the average lifetime was calculated from literature.[4]

c(Ni^{2+})	τ_1 (ns)	B_1	τ_2 (ns)	B_2	τ_3 (ns)	B_3	$\langle\tau\rangle$ (ns)
**0 mM**	1.74	0.305	14.3	0.0557	80.7	0.0104	37.0
**0.005 mM**	1.65	0.279	12.7	0.0492	78.7	0.00720	32.2
**0.01 mM**	1.44	0.299	11.5	0.0564	74.6	0.00657	28.5
**0.02 mM**	1.45	0.289	9.98	0.0570	59.6	0.00734	22.7
**0.04 mM**	1.21	0.300	7.61	0.0637	44.5	0.00912	17.7

Supplementary Table 7. Kinetic analysis of emission decay for InP/ZnS-S QDs (525 nm, 15 min) with increasing H_2A concentration. Further addition of H_2A was ceased at 3 mM, as the emission lifetime of QDs at this stage is close to the detection limit of the instrument, and the quenching of PL was too pronounced for precise measurements. The amounts of H_2A applied were far below photocatalytic conditions (0.2 M H_2A). Note that H_2A is consumed in the reaction and an excess amount is required for a long time reaction. For the measurement of emission spectroscopy, the pH of all solutions was adjusted to 4.5, and the excitation wavelength was 406 nm. The decay traces for the samples were well fitted with a triple-exponential function and the average lifetime was calculated from literature.[4]

**c(H_2A)**	**τ_1 (ns)**	**B_1**	**τ_2 (ns)**	**B_2**	**τ_3 (ns)**	**B_3**	**$\langle\tau\rangle$ (ns)**
**0 mM**	1.91	0.261	14.6	0.0551	80.3	0.0110	38.2
**0.04 mM**	1.62	0.340	12.1	0.0545	71.7	0.00789	27.8
**0.1 mM**	1.52	0.350	11.9	0.0484	67.7	0.00557	22.3
**0.2 mM**	1.14	0.407	7.36	0.0584	39.2	0.00816	13.4
**0.4 mM**	0.942	0.342	5.89	0.0616	33.8	0.00722	11.5
**0.8 mM**	0.924	0.392	5.29	0.0537	25.9	0.00688	7.83
**1.6 mM**	0.746	0.445	4.04	0.0507	17.7	0.00568	4.47
**3.0 mM**	0.535	0.543	2.67	0.0480	10.6	0.00653	2.52

Supplementary Table 8. Fitting results of EIS data of InP/ZnS QDs capped with different ligands. Larger errors were due to the very large resistance in the systems with InP/ZnS-OLA QDs and blank and samples under dark conditions.

	QDs	$R_{solution}$ (Ω)	Error%	R_{ct} (Ω)	Error%	$C_{electrode}$ (F)	Error%
**hν**	InP/ZnS-S	138.9	16.47	23.21 K	14.10	3.216	9.34
	InP/ZnS-MPA	122.6	12.16	77.74 K	9.91	3.021	11.73
	InP/ZnS-OLA	227.5	53.17	1.388 M	184.62	1.693	28.51
	Blank	139.8	10.66	615.2 K	98.10	2.016	7.60
**dark**	InP/ZnS-S	138.1	12.70	1.838 M	87.33	1.752	5.81
	InP/ZnS-MPA	117.0	11.52	1.391 M	100.22	1.606	7.99
	InP/ZnS-OLA	264.1	64.59	1.156 M	180.08	1.564	23.30
	Blank	137.9	10.36	642.3 K	92.17	1.986	7.30

Supplementary Notes

Supplementary Note 1: Purification of InP and InP/ZnS QD with short excitonic absorption peaks.

It should be pointed out that when more zinc(II) iodide was added to obtain QDs with smaller size (480 nm and especially 440 nm), the size distribution of the QDs became broader. As a result, a certain amount of QDs with smaller size did not precipitate from the system, but remained in the supernatant during purification. These QDs should be discarded as well.[5] In addition, we also found that with higher amounts of zinc(II) iodide added, the unreacted raw material maintained a similar color to the QDs itself. This renders the purification process more difficult, because the precipitation of QDs is often calibrated through the color changes of the supernatant.

Supplementary Note 2: Influence of pH and other parameters on QDs in photocatalytic media.

In addition to the description in the main text, we furthermore increased the acidity of the solution gradually (by decreasing the pH in steps of 0.5 units) and we noticed that precipitation set in only after the pH dropped to 2.5 (Supplementary Fig. 19). This value is much lower than the pK_a of H_2S/HS^- (6.9-7.0). This result agrees well with a previous study reporting on the precipitation of thiol capped Cd-based QDs by X. Peng's group.[6] The pK_a of –S/–SH for thiol was around 10, but ligand removal (and precipitation of QDs) occurred at pH 4-6 for different series of QDs. Both results show that once the ligands are bound on the surface of QDs, their original nature can be drastically changed.

When the pH was adjusted to 4.5 in zeta potential measurements (Supplementary Table 4), the absolute zeta potential of the solution decreased a little (from -36.1 to -31.2 mV). This zeta potential value is sufficiently negative to stabilize the colloidal solution and indicates that most of S^{2-} ligands were retained, although a small proportion may be protonated. Further addition of Ni^{2+} into the solution decreased the zeta potential (-26.9 mV), in line with the expected adsorption of Ni^{2+} on the surface of S^{2-} capped QDs.[7] Introduction of H_2A/NaHA decreased the zeta potential of the system as well, probably due to adsorption of a small amount of H_2A/NaHA on the surface of the QDs.[8] Although H_2A/NaHA probably does not interact strongly with QDs, the large amount of H_2A/NaHA (0.2 M) might increase the interaction.

Supplementary Note 3: DFT calculation results of InP with different surface states.

As shown in the difference charge density map (Supplementary Fig. S26), the passivation by S ligands leads to a charge redistribution between the atomic In and S layers. Obvious charge transfer between In and S atoms implies that S ligands chemically bond with the In atoms on the top of the InP surface and that electron delivery channels are formed at the interface. These formed electron delivery channels can thus enhance the transfer of photoinduced carriers.

Based on the band structure of InP-H, it is noticeable that the introduction of S^{2-} and the intrinsically incomplete InP surface both yield defect states (Supplementary Fig. 27a-c). Defect states related to the intrinsic InP QDs could be removed by addition of ZnS on the surface of InP (Supplementary Fig. 27d), which is in agreement with our experimental results. It should be taken into account that, due to the simplified model applied for the present system and due to the limitations of the DFT calculation method, the results in Supplementary Fig. S27 mainly confirm the existence of two different defect types. However, the calculated positions of the energy levels may be quite different from their actual positions.

Given the probable inaccuracy of DFT calculations, we also carried out further experiments, such as steady state and time resolved spectroscopy, to study the specific energy level of the defect states. However, such attempts were not successful. For example, we did not observe any trap-state emission of InP based QDs, neither in the visible light nor in the near infrared light range.[8]

Considering the diversity and complexity of the surface states of QDs,[9] the defect states may have a wide energy level distribution, and to date there is still a lack of widely recognized methods for the determination of QD defect energy levels. Therefore, the energy level positions of either intrinsic defects or surface S^{2-} ligands were indicated indistinctly here with several line symbols (Supplementary Fig. 28), which is a common practice in the description of defect states of QDs.

Supplementary Methods

Supplementary Method 1: General information.

$InCl_3$ (99.99%), $ZnCl_2$ (98%), ZnI_2 (98%), tris(diethylamino)phosphine (97%), sulfur (S, 99%), ascorbic acid (H_2A, 99%), sodium ascorbate (NaHA, 99%), oleylamine (OLA, 90%), trioctylphosphine (TOP, 90%), $NiCl_2$ (98%) and N-methylformamide (NMF, 99%) were obtained from Sigma-Aldrich; $Na_2S\cdot 9H_2O$ (99.99%) and formamide (FA, 99%) were obtained from Alfa-Aesar. Hexane (HEX, 99%) was obtained from Thommen Furler, and ethanol (99.7%) and acetone (99.5%) were obtained from Reuss Chemie. Reagents were all used as received. Deionized water was used throughout the experiments.

Transmission electron microscopy (TEM) and high-resolution transmission electron microscopy (HRTEM) was performed on a JEM 2100F microscope (operated at an accelerating voltage of 200 kV). Aberration-corrected scanning transmission electron microscopy (STEM) was performed on a Titan Themis microscope operated at 300 kV in high angle annular dark filed (HAADF) mode. Powder X-ray diffraction patterns were recorded on a STOE STADI P powder diffractometer, operated in transmission mode, with Cu-K_α radiation at λ = 1.54056 Å. UV-Vis absorption spectra were recorded on a PerkinElmer Lambda 650S UV/VIS spectrophotometer. Steady-state and transient emission spectroscopy (time correlated single photon counting) was performed on a Horiba Fluorolog3 spectrofluorometer. Elemental analysis data were obtained from Inductively Coupled Plasma Optical Emission Spectrometry (ICP-OES, Varian 710-OES, USA). SEM-EDS investigations were performed on a Hitachi S4800 instrument. FT-IR spectra were recorded on a Bruker Vertex 70 spectrometer. Thermogravimetric analysis was carried out on a NETZSCH STA 449 F3 Jupiter. Zeta potential values were measured using a Zetasizer Nano-ZS90 (Malvern Instruments). Cyclic voltammetry experiments were performed under nitrogen atmosphere on an Autolab Potentiostat Galvanostat electrochemical workstation with a one-compartment three electrode cell, using glassy carbon working electrodes, a Pt counter electrode and an Ag pseudo-reference electrode. Femtosecond transient absorption experiments (fs-TA) were conducted using a home-built femtosecond broadband pump–probe setup with time resolution around 100 fs as described previously in detail.[10,11] EPR spectra were measured on an EPR spectrometer (Bruker-E 500) at room temperature and 9.868 GHz. ^{13}C NMR spectra were recorded on a

Bruker Avance II 400 MHz NMR Spectrometer. The pH value was measured with a Model pHS-3C meter (Mettler Toledo FE20).

Surface photovoltage (SPV) measurements were recorded on a CEL-SPS1000 (Au-Light Co., Ltd) on the basis of the lock-in amplifier, including a lock-in amplifier (SR830, Stanford research systems, Inc.), monochromatic-light, a light chopper (SR540, Stanford research systems, Inc.), and a chamber. Monochromatic light was generated from a 150 W xenon lamp (CEL-S150) by a monochromator (CEL-IS151). QD powders were used directly and we constructed the photovoltaic cell as a sandwich-like architecture of ITO-sample-ITO. During the measurement, we aimed to standardize the samples by using the same sample mass on the same area of ITO.

Photoelectrochemical tests were carried out on a Zahner Zennium electrochemical workstation. A three electrode cell was used with Ag/AgCl (in saturated KCl solution) and Pt wire as reference and counter electrodes and 0.2 M H_2A (pH 4.5) as electrolyte. The working electrode was prepared by drop-casting of 10 μL of prepared stock solution (with 10 mM InP (molecular unit) concentration and Nafion weight percentage of 0.5%) on the surface of FTO (with an exposed area of 0.20 cm^2). The stock solution was prepared by mixing different QD solutions with a certain amount of Nafion solution (5%). For QDs with OLA, MPA, and S^{2-} ligands, acetone/hexane mixture, water, and NMF were used, respectively, as solvents to generate a clear stock solution. For EIS tests, a frequency range from 0.1 to 10,000 Hz was selected with the AC voltage amplitude of 5 mV.

All spin-polarized DFT calculations were carried out using the Vienna Ab Intio Simulation Package (VASP)[12] with a cut-off energy of 450 eV. The exchange and correlation potential was described by the generalized gradient approximation of the Perdew-Burke-Ernzerhof (PBE) functional.[13] The geometry optimizations of periodic models were terminated when the convergence criteria of energy and force were reduced below 1×10^{-4} eV and 0.05 eV/Å, respectively. Spin polarization and dipole correction were applied for calculating electronic properties of the optimized configurations. The Brillouin zones were sampled with 4×4×1 and 8×8×1 k points for the structure optimizations and electronic properties, respectively. As shown in Supplementary Fig. 25a, the (1×1) period InP (001) slab model containing 10 atomic layers of In and 9 atomic layers of P (19 layers of InP) with an interval of 10 Å was constructed referring to a previous study,[14] where

the surface was passivated by pseudo-hydrogen atoms (with charge 1.25 e). The structure of InP-S was built based on the (1×1) period InP (001) slab model where the top layer was passivated with S ligands (Supplementary Fig. 25b). As for the structure of InP with intrinsic defects, a (2×2) period InP (001) slab model was employed and the coverage of In vacancy was 1/4 ML (Supplementary Fig. 25c). A layer of ZnS (001) was combined with InP to mimic the structure of InP/ZnS (Supplementary Fig. 25d).

Supplementary Method 2: Calculation of InP and InP/ZnS QD concentrations.

As it is difficult to find a straightforward and appropriate empirical formula to directly calculate the QD concentration of InP QDs in contrast to Cd-based QDs,[15] we first derived the InP unit/molecule concentration from absorption spectra. The basic assumption is that the intrinsic absorption coefficient of colloidal nanocrystals is close to that of bulk materials in the short wavelength range. Knowing the intrinsic absorption coefficient of bulk InP, we can use these values to calculate the molecular and mass concentration of InP QD solutions.[16] Different absorption wavelengths of the InP QDs were used for the calculation, in particular 310 nm,[17,18] 350 nm,[19] and 413 nm.[16] In our case, we have selected 413 nm for concentration estimations to minimize the influence of ZnS, which may display absorption in the shorter wavelength range. Calculations were performed as follows:[16]

The intrinsic absorption coefficient is given by:

$$\mu_{i,th} = \frac{4\pi nk\,|f_{\mathrm{LF}}|^2}{n_s \lambda}$$ Supplementary Equation 1

n and k are the real and imaginary part of the refractive index of bulk zinc-blende InP, and n_S is the refractive index of the solvent. The local field factor f_{LF} is given by:

$$|f_{\mathrm{LF}}|^2 = \frac{9n_s^4}{\left(n^2 - k^2 + 2n_s^2\right)^2 + 4\left(nk\right)^2}$$ Supplementary Equation 2

Where $\lambda = 413$ nm, $n = 4.395$, $k = 1.247$ were taken from literature,[16] $n_s = 1.387$ for hexane and $n_s = 1.342$ for water at 413 nm were obtained from other reports.[20,21] The amount of InP unit/molecule n_{cuvette} is calculated as:

$$n_{\text{cuvette}} = \frac{A\ln 10}{\mu_{i,th}L} \times \frac{V_{\text{cuvette}}}{V_{\text{m}}}$$ Supplementary Equation 3

where L is the cuvette length (m), V_{m} is the InP molar volume, and A is the measured absorbance of QDs at 413 nm (A should be at a suitable value range). Here, L is 0.01 m and V_{m} is 3.0×10^{-5} m^3 mol^{-1}.[16]

The InP unit /molecule concentration c_{molecule} is then obtained from the following equation:

$$c_{\text{molecule}} = \frac{n_{\text{cuvette}}}{V_{\text{cuvette}}} = \frac{A\ln 10}{\mu_{i,th}L} \times \frac{1}{V_{\text{m}}}$$ Supplementary Equation 4

For hexane as solvent, $\left|f_{\text{LF}}\right|^2 = 0.0567$, $\mu_{i,th} = 6.82\times 10^6\,\text{m}^{-1}$

$$c_{\text{molecule}} = A\times 1.13\ \text{mol m}^{-3} = A\times 1.13\times 10^{-3}\ \text{mol L}^{-1}$$ Supplementary Equation 5

For water as solvent, $\left|f_{\text{LF}}\right|^2 = 0.0506$, $\mu_{i,th} = 6.29\times 10^6\,\text{m}^{-1}$

$$c_{\text{molecule}} = A\times 1.22\ \text{mol m}^{-3} = A\times 1.22\times 10^{-3}\ \text{mol L}^{-1}$$ Supplementary Equation 6

To further calculate the concentration of QDs, the number of molecules per QD, N, was estimated. Taking InP/ZnS QDs (525 nm) for instance, we here assume that the QDs are spherical. The corresponding diameter of QDs was averaged at 2.7 nm from the TEM statistics (see Supplementary Fig. 8). The number of InP molecules per QD was then calculated as follows:[22]

$$N = \frac{\pi n R^3}{6a_0^3}$$ Supplementary Equation 7

R is the diameter of the QDs, a_0 is the lattice constant of bulk InP with zinc blende structure (0.583 nm),[23] and n is the number of molecules in per unit cell of InP (n = 4). For R = 2.7 nm we thus calculated N = 208. Accordingly,

$$c_{\text{QDs}} = c_{\text{molecule}} / 208$$ Supplementary Equation 8

Supplementary Method 3: Preparation and hydrogen evolution of reference CdSe QDs.

Two methods were applied to synthesize CdSe QDs with comparable absorption peaks and sizes to InP QDs (525 nm). The first route via octadecene (ODE) was modified from literature[24,25] and is referred to here as

CdSe-A. Briefly, 0.4 mmol of CdO, 0.5 mL of oleic acid (OA), 1.5 mL OLA, and 8 mL of ODE were mixed in a 50 mL three-neck flask, and the mixture was heated to 240 °C. Then 1 mL of ODE solution containing 100 μL TOP-Se (2 M) was injected. The reaction was kept for 30 s and cooled down to room temperature quickly. The second route (here: CdSe-B) via trioctylphosphine oxide (TOPO) was taken without changes from literature.[26] Purification and ligand exchange for CdSe QDs were conducted as described for InP QDs in the Methods in the main text.

The photocatalytic hydrogen evolution experiment was carried out at a concentration where InP QDs and CdSe QDs in the reaction system had the same absorbance at 525 nm (i.e. the wavelength of the LED light source). The remaining hydrogen evolution conditions were the same. We emphasize that these control experiments are just for reference purposes and do not permit absolute conclusions, because the optimal conditions and the most suitable catalysts for hydrogen evolution may differ for different QD types.

Supplementary Method 4: Calculation of the TON value and hydrogen evolution rate of InP/ZnS QDs.

The TON value based on QDs and hydrogen evolution rate was calculated from Supplementary Fig. 13a, where 1.23 mmol of H_2 was generated within 64.5 h. Therefore, the TON based on QDs should be 1.23 mmol/9.62 nmol, i.e. 128,000. The corresponding TON based on InP molecules was further calculated to be 1.23 mmol/2.0 μmol, i.e. 615. On the other hand, the amount of Ni^{2+} added in the solution was 210 nmol, so that the TON value based on Ni was 5,860.

We also estimated the hydrogen evolution rate based on QD mass. The mass of InP/ZnS QDs was determined as 0.42 ± 0.03 mg in a typical photocatalytic experiment from four parallel gravimetric measurements (the amounts of QDs were proportionally increased for precise weighing). Accordingly, the hydrogen evolution rate is 45 $mmol \cdot g^{-1} \cdot h^{-1}$.

Besides, we calculated the mass of QDs from the molar amount of InP QDs, which was derived from the optical absorbance of QDs. For a hydrogen evolution system with InP/ZnS QDs containing 2 μmol of InP molecules, the corresponding weight of InP is 146 mg/μmol × 2 μmol, i.e. 0.292 mg. From ICP results, the weight percentage of InP is 73.1% in InP/ZnS QDs, so that the weight of InP/ZnS QDs is calculated to be 0.40

mg in the reaction system. This value is similar to that from gravimetric analyses, demonstrating that the calculation of concentrations in Supplementary Method 2 is reasonable.

Supplementary Method 5: Measurement of apparent and internal quantum efficiency.

For monochromatic LED light sources (λ = 465 nm), the accurate illumination power for a certain area of the reaction mixture (1 cm^2) was measured using a digital photodiode power meter (Newport, model 842-PE). Photocatalytic hydrogen evolution was performed in a custom-made spectro-cell with a total volume of 7 mL and a path-length of 1 cm. The cuvette was filled with 3 mL of a solution containing 525 nm InP/ZnS QDs (1.6 μM) and $NiCl_2 \cdot 6H_2O$ (0.035 mM) at pH 4.5. The system was then sealed and deoxygenated with argon for 15 min. Under constant stirring, the solution was irradiated at the same place and area (1 cm^2) as applied for power measurements above. The number of absorbed photons was calculated from the illumination power and the absorbance of QDs from the reaction solution, while the molar amount of H_2 was quantitatively analyzed by GC.

Based on the average of four experiments, the illumination power (*P*) was determined as 66 ± 1 mW, the absorbance (*A*) of the reaction solution in 465 nm was 0.158 ± 0.002, and hydrogen evolution over 30 min was 435 ± 40 μL (1.94 × 10^{-5} mol). Consequently, the internal quantum efficiency was calculated as follows:[1]

Amount of hydrogen molecules generated per second:

$$k_{H_2} = \frac{n_{H_2}}{t} = \frac{1.94\times10^{-5}\,\text{mol}}{1800\text{s}} = 1.08\times10^{-8}\,\text{mol s}^{-1} = 6.50\times10^{15}\ \text{s}^{-1}$$ Supplementary Equation 9

Amount of incident photons per second:

$$q_{\text{incident photons}} = \frac{P\lambda}{hc} = \frac{0.066\ \text{W}\ \times 465\ \text{nm}}{6.626\times10^{-34}\,\text{J}\cdot\text{s}\ \times 3.0\times10^{8}\,\text{m s}^{-1}} = 1.54\times10^{17}\,\text{s}^{-1}$$ Supplementary Equation 10

Amount of absorbed photons per second:

$$q_{\text{absorbed photons}} = q_{\text{incident photons}} \times\left(1-10^{-A}\right) = 1.54\times10^{17}\,\text{s}^{-1}\times\left(1-10^{-0.158}\right) = 4.70\times10^{16}\,\text{s}^{-1}$$

Supplementary Equation 11

Apparent quantum efficiency (AQY):

$$\Phi_{H_2}\left(\text{AQY}\right) = \frac{2k_{H_2}}{q_{\text{incident photons}}}\times100\% = \frac{2\times6.50\times10^{15}\,\text{s}^{-1}}{1.54\times10^{17}\,\text{s}^{-1}}\times100\% = 8.44\%$$ Supplementary Equation 12

Internal quantum efficiency (IQY):

$$\Phi_{H_2}(\mathrm{IQY}) = \frac{2k_{H_2}}{q_{\text{absorbed photons}}} \times 100\% = \frac{2\times 6.50\times 10^{15}\,\mathrm{s}^{-1}}{4.70\times 10^{16}\,\mathrm{s}^{-1}} \times 100\% = 27.7\%$$ Supplementary Equation 13

Accordingly, we also determined other AQY/IQY of the system at several other wavelengths, and the results are listed in Supplementary Table 3. In addition, good correlation of the AQY values with the absorption spectra of InP/ZnS QDs is shown in Supplementary Fig. 14.

Supplementary Method 6: Kinetics analysis of charge transfer for InP/ZnS QD with Ni^{2+} and H_2A.

Kinetic analysis of the charge carrier transfer rate was carried out according to Supplementary Equation 14 as reported in the literature.[27,28] $<\tau>$ and $<\tau_0>$ are the average lifetime of QDs in the presence/absence of Ni^{2+} and H_2A, which was obtained from Supplementary Table 6 and Supplementary Table 7, and c represents the corresponding Ni^{2+} and H_2A concentration.

$$k_{ct} = \left(1/<\tau> - 1/<\tau_0>\right)/c$$ Supplementary Equation 14

Furthermore, the charge transfer time from QDs to Ni^{2+} and H_2A in the photocatalytic system was calculated as well. Charge transfer from QDs to Ni^{2+} can be well deduced by the linear relation in the inset of Figure 4c of the main text (7.64×10^8 s^{-1} mM^{-1}). For a Ni^{2+} concentration of 0.035 mM, the charge transfer rate is 2.67×10^7 s^{-1}, so that the electron transfer time is 37.5 ns. Moreover, the charge transfer from QDs to H_2A/NaHA was evaluated in a similar way. It is noteworthy that H_2A/NaHA is applied in excess under the given reaction conditions. However, if we add a large amount of H_2A/NaHA into the QD solution, their PL was entirely quenched during measurements, so that the dynamic decay of PL could not be determined due to the limits of the PL intensity and instrument response function (IRF). Therefore, we here calculated the hole transfer rate from QDs to H_2A/NaHA for a medium concentration of H_2A/NaHA (30 mM) with the average lifetime of 1.13 ns at 30 mM H_2A/NaHA (τ_1 = 0.2625 ns, B_1= 0.08331; τ_2 = 1.341 ns, B_2= 0.006171; τ_3 = 7.147 ns, B_3= 0.0004033). Correspondingly, the hole transfer time was determined as 1.16 ns.

Supplementary Method 7: Ligand exchange of InP/ZnS QDs with MPA and MUA.

To demonstrate the effect of S^{2-} ligands on the photocatalytic performance, reference InP/ZnS QDs with

the commonly used thiol ligands MPA and MUA were prepared. The solutions for ligand exchange with MPA and MUA were prepared similarly as to S^{2-} capping by mixing QD solutions in HEX with MPA and MUA in NMF. As MPA and MUA are acidic, an according amount of base, such as tetramethylammonium hydroxide pentahydrate (TMAH), should be added first.[5] In a typical process, 1 mL of InP or InP/ZnS QDs (about 3-5 mg mL^{-1}) in HEX was mixed with 1 mL of 0.1 M MPA/MUA and 0.2 M TMAH in NMF. The mixture was kept stirring at room temperature until a total phase transfer of QDs from HEX to NMF was achieved. The washing process for MPA and MUA was the same as applied for S^{2-} capping, while higher acetone amounts were required to precipitate the QDs. The photocatalytic hydrogen evolution experiment was carried out with the same concentration of QDs (1.6 μM) with different ligands.

Supplementary Method 8: Ligand exchange of InP/ZnS QDs with Cl^- and PO_4^{3-}.

To further show the significance of S^{2-} ligands, two other inorganic ligands, namely Cl^- and PO_4^{3-}, were chosen here for comparison because of their feasibility for ligand exchange and their stability in ambient environments.[29,30] It should be pointed out that the use of NaCl or KCl did not lead to a successful phase transfer of QDs into water, so that $InCl_3$ and $ZnCl_2$ were used as Cl^- sources instead. This phenomenon is very similar to previous reports.[29] In a typical process, 1 mL of InP or InP/ZnS QDs (about 3-5 mg mL^{-1}) in HEX was mixed with 1 mL of 0.025 M $Na_3PO_4 \cdot 12H_2O$ in FA, or 0.05 M $ZnCl_2$/$InCl_3$ in NMF. The mixture was kept under stirring until a total phase transfer of QDs from HEX to FA/NMF was achieved, but here more time or higher temperature are needed for a total phase transfer of the QDs in contrast to S^{2-}. The washing process for Cl^- and PO_4^{3-} was the same as that applied for S^{2-} capping. The photocatalytic hydrogen evolution experiment was carried out with the same concentration of QDs (1.6 μM) with different ligands. For photocatalytic experiment comparison with QDs-Cl, we finally used QDs obtained from ligand exchange with $InCl_3$, as the hydrogen evolution rate for $InCl_3$ is better than that of QDs after ligand exchange with $ZnCl_2$.

Supplementary References.

1. Han, Z., Qiu, F., Eisenberg, R., Holland, P. L. & Krauss, T. D. Robust Photogeneration of H_2 in Water Using Semiconductor Nanocrystals and a Nickel Catalyst. *Science* **338,** 1321–1324 (2012).
2. Indra, A. *et al.* Nickel as a co-catalyst for photocatalytic hydrogen evolution on graphitic-carbon nitride (sg-CN): what is the nature of the active species? *Chem. Commun.* **52,** 104–107 (2016).
3. Lian, S., Kodaimati, M. S., Dolzhnikov, D. S., Calzada, R. & Weiss, E. A. Powering a CO_2 Reduction Catalyst with Visible Light through Multiple Sub-picosecond Electron Transfers from a Quantum Dot. *J. Am. Chem. Soc.* **139,** 8931–8938 (2017).
4. James, D. R., Liu, Y.-S., Mayo, P. de & Ware, W. R. Distributions of fluorescence lifetimes: consequences for the photophysics of molecules adsorbed on surfaces. *Chem. Phys. Lett.* **120,** 460–465 (1985).
5. Ramasamy, P., Kim, B., Lee, M.-S. & Lee, J.-S. Beneficial effects of water in the colloidal synthesis of InP/ZnS core-shell quantum dots for optoelectronic applications. *Nanoscale* **8,** 17159–17168 (2016).
6. Aldana, J., Lavelle, N., Wang, Y. & Peng, X. Size-Dependent Dissociation pH of Thiolate Ligands from Cadmium Chalcogenide Nanocrystals. *J. Am. Chem. Soc.* **127,** 2496–2504 (2005).
7. Nag, A. *et al.* Effect of Metal Ions on Photoluminescence, Charge Transport, Magnetic and Catalytic Properties of All-Inorganic Colloidal Nanocrystals and Nanocrystal Solids. *J. Am. Chem. Soc.* **134,** 13604–13615 (2012).
8. Ye, Y. *et al.* Charge-Transfer Dynamics Promoted by Hole Trap States in CdSe Quantum Dots–Ni^{2+} Photocatalytic System. *J. Phys. Chem. C* **121,** 17112–17120 (2017).
9. Boles, M. A., Ling, D., Hyeon, T. & Talapin, D. V. The surface science of nanocrystals. *Nat. Mater.* **15,** 141–153 (2016).
10. Wang, X. *et al.* Odd–Even Effect of Thiophene Chain Lengths on Excited State Properties in Oligo(thienyl ethynylene)-Cored Chromophores. *J. Phys. Chem. C* **121,** 7659–7666 (2017).
11. Long, S. *et al.* Energy transfer and spectroscopic characterization of a perylenetetracarboxylic diimide (PDI) hexamer. *Phys. Chem. Chem. Phys.* **17,** 18567–18576 (2015).
12. Kresse, G. & Furthmüller, J. Efficient iterative schemes for ab initio total-energy calculations using a plane-wave basis set. *Phys. Rev. B* **54,** 11169–11186 (1996).
13. Perdew, J. P., Burke, K. & Ernzerhof, M. Generalized Gradient Approximation Made Simple. *Phys. Rev. Lett.* **77,** 3865–3868 (1996).
14. KC, S. *et al.* Electronic properties of InP (001)/HfO_2 (001) interface: Band offsets and oxygen dependence. *J. Appl. Phys.* **115,** 23703 (2014).
15. Yu, W. W., Qu, L., Guo, W. & Peng, X. Experimental Determination of the Extinction Coefficient of CdTe, CdSe, and CdS Nanocrystals. *Chem. Mater.* **15,** 2854–2860 (2003).
16. Tessier, M. D., Dupont, D., Nolf, K. de, Roo, J. de & Hens, Z. Economic and Size-Tunable Synthesis of InP/ZnE (E = S, Se) Colloidal Quantum Dots. *Chem. Mater.* **27,** 4893–4898 (2015).
17. Xie, L., Harris, D. K., Bawendi, M. G. & Jensen, K. F. Effect of Trace Water on the Growth of Indium Phosphide Quantum Dots. *Chem. Mater.* **27,** 5058–5063 (2015).
18. Xie, L. *et al.* Characterization of Indium Phosphide Quantum Dot Growth Intermediates Using MALDI-TOF Mass Spectrometry. *J. Am. Chem. Soc.* **138,** 13469–13472 (2016).
19. Buffard, A. *et al.* Mechanistic Insight and Optimization of InP Nanocrystals Synthesized with Aminophosphines. *Chem. Mater.* **28,** 5925–5934 (2016).
20. Daimon, M. & Masumura, A. Measurement of the refractive index of distilled water from the near-infrared

region to the ultraviolet region. *Appl. Opt.* **46,** 3811–3820 (2007).

21. Kozma, I. Z., Krok, P. & Riedle, E. Direct measurement of the group-velocity mismatch and derivation of the refractive-index dispersion for a variety of solvents in the ultraviolet. *J. Opt. Soc. Am. B* **22,** 1479–1483 (2005).
22. Rama Krishna, M. V. & Friesner, R. A. Quantum confinement effects in semiconductor clusters. *J. Chem. Phys.* **95,** 8309–8322 (1991).
23. Fu, H. & Zunger, A. InP quantum dots: Electronic structure, surface effects, and the redshifted emission. *Phys. Rev. B* **56,** 1496–1508 (1997).
24. Wang, P., Zhang, J., He, H., Xu, X. & Jin, Y. The important role of surface ligand on CdSe/CdS core/shell nanocrystals in affecting the efficiency of H_2 photogeneration from water. *Nanoscale* **7,** 5767–5775 (2015).
25. Zhu, H., Song, N. & Lian, T. Controlling Charge Separation and Recombination Rates in CdSe/ZnS Type I Core−Shell Quantum Dots by Shell Thicknesses. *J. Am. Chem. Soc.* **132,** 15038–15045 (2010).
26. Zhu, H., Song, N., Lv, H., Hill, C. L. & Lian, T. Near unity quantum yield of light-driven redox mediator reduction and efficient H_2 generation using colloidal nanorod heterostructures. *J. Am. Chem. Soc.* **134,** 11701–11708 (2012).
27. Kongkanand, A., Tvrdy, K., Takechi, K., Kuno, M. & Kamat, P. V. Quantum dot solar cells. Tuning photoresponse through size and shape control of CdSe-TiO_2 architecture. *J. Am. Chem. Soc.* **130,** 4007–4015 (2008).
28. Yun, H. J., Paik, T., Edley, M. E., Baxter, J. B. & Murray, C. B. Enhanced Charge Transfer Kinetics of CdSe Quantum Dot-Sensitized Solar Cell by Inorganic Ligand Exchange Treatments. *ACS Appl. Mater. Interfaces* **6,** 3721–3728 (2014).
29. Dirin, D. N. *et al.* Lead halide perovskites and other metal halide complexes as inorganic capping ligands for colloidal nanocrystals. *J. Am. Chem. Soc.* **136,** 6550–6553 (2014).
30. Huang, J. *et al.* Surface functionalization of semiconductor and oxide nanocrystals with small inorganic oxoanions (PO_4^{3-}, MoO_4^{2-}) and polyoxometalate ligands. *ACS Nano* **8,** 9388–9402 (2014).
